# The resistance mechanisms of bacteria against ciprofloxacin and new approaches for enhancing the efficacy of this antibiotic

**DOI:** 10.3389/fpubh.2022.1025633

**Published:** 2022-12-21

**Authors:** Aref Shariati, Maniya Arshadi, Mohammad Ali Khosrojerdi, Mostafa Abedinzadeh, Mahsa Ganjalishahi, Abbas Maleki, Mohsen Heidary, Saeed Khoshnood

**Affiliations:** ^1^Molecular and Medicine Research Center, Khomein University of Medical Sciences, Khomein, Iran; ^2^Infectious and Tropical Diseases Research Center, Health Research Institute, Ahvaz Jundishapur University of Medical Sciences, Ahvaz, Iran; ^3^Department of Microbiology, School of Medicine, Ahvaz Jundishapur University of Medical Sciences, Ahvaz, Iran; ^4^Student Research Committee, Sabzevar University of Medical Sciences, Sabzevar, Iran; ^5^Clinical Microbiology Research Center, Ilam University of Medical Sciences, Ilam, Iran; ^6^Department of Laboratory Sciences, School of Paramedical Sciences, Sabzevar University of Medical Sciences, Sabzevar, Iran; ^7^Cellular and Molecular Research Center, Sabzevar University of Medical Sciences, Sabzevar, Iran; ^8^Student Research Committee, Ilam University of Medical Sciences, Ilam, Iran

**Keywords:** review, ciprofloxacin, resistance, new approach, antibacterial agents

## Abstract

For around three decades, the fluoroquinolone (FQ) antibiotic ciprofloxacin has been used to treat a range of diseases, including chronic otorrhea, endocarditis, lower respiratory tract, gastrointestinal, skin and soft tissue, and urinary tract infections. Ciprofloxacin's main mode of action is to stop DNA replication by blocking the A subunit of DNA gyrase and having an extra impact on the substances in cell walls. Available in intravenous and oral formulations, ciprofloxacin reaches therapeutic concentrations in the majority of tissues and bodily fluids with a low possibility for side effects. Despite the outstanding qualities of this antibiotic, *Salmonella typhi, Staphylococcus aureus, Escherichia coli*, and *Pseudomonas aeruginosa* have all shown an increase in ciprofloxacin resistance over time. The rise of infections that are resistant to ciprofloxacin shows that new pharmacological synergisms and derivatives are required. To this end, ciprofloxacin may be more effective against the biofilm community of microorganisms and multi-drug resistant isolates when combined with a variety of antibacterial agents, such as antibiotics from various classes, nanoparticles, natural products, bacteriophages, and photodynamic therapy. This review focuses on the resistance mechanisms of bacteria against ciprofloxacin and new approaches for enhancing its efficacy.

## Introduction

A member of the fluoroquinolone (FQ) family of antibiotics, ciprofloxacin can be used to treat a variety of Gram-positive and Gram-negative bacteria. FQs regulate bacterial DNA supercoiling, a procedure necessary for DNA replication, recombination, and repair, by binding to and inhibiting DNA gyrase enzymes. The United States Food and Drug Administration (FDA) has given the drug approval for the treatment of gastrointestinal and lower respiratory tract infections, anthrax, plague, salmonellosis, skin, bone, and joint infections, prostatitis, typhoid fever, and sexually transmitted infections like gonorrhea and chancroid. It has also been recommended by World Health Organization (WHO) for treating tuberculosis (TB) as the second-line treatment for multidrug-resistant (MDR) TB (1, 2).

Nonetheless, there are increasing reports of ciprofloxacin resistance in *Bacillus anthracis, Pseudomonas aeruginosa, Neisseria gonorrhoeae, Enterococci, Escherichia coli*, and *Klebsiella pneumoniae* (3, 4). The resistance could develop by efflux pumps or mutations in DNA gyrase genes (*gyrA*) (3, 5). Ciprofloxacin can also be used in the treatment of malaria (6). In this regard, the review mainly concentrated on the various properties of ciprofloxacin, its clinical applications for the treatment of different microbial infections, and bacterial resistance mechanisms to this antibiotic, as well as new strategies for enhancing ciprofloxacin efficacy against MDR bacteria.

## Ciprofloxacin characteristics

### Structure of drug

**Figure 1:**
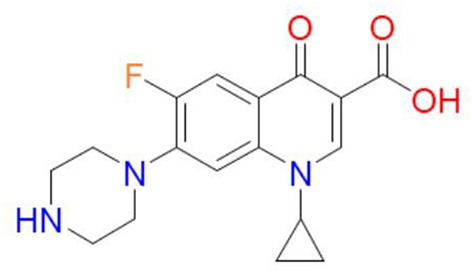
Chemical structure of ciprofloxacin (9).

One-cyclopropyl-6-fluoro-4-oxo-7-(piperazine-1-yl)-1, 4-dihydroquinoline-3-carboxylic acid is the molecular name for the antibiotic (6). Its molecular weight is 331.34 g/mol and its chemical formula is C_17_H_18_FN_3_O_3_ (7). A quinolone, quinolin-4(1H)-one is the name of the antibiotic, and it has the functional groups cyclopropyl, carboxylic acid, fluoro, and piperazin-1-yl at positions 1, 3, 6, and 7, respectively (Figure 1) (7). The fluorine group at position C-6 and the piperazine group cause the expansion of the antimicrobial spectrum of ciprofloxacin. The piperazine group, also found in cefoperazone and piperacillin, increases ciprofloxacin activity against *Pseudomonas*. The cyclopropyl group is related to the high antibacterial activity of ciprofloxacin (8).

### Pharmacokinetics and pharmacodynamics

The pharmacokinetic profile of ciprofloxacin has been investigated for absorption, distribution, metabolism, and clearance. Studies have been performed by testing healthy and patient volunteers. Ciprofloxacin is absorbed fast and well and penetrates the tissues very well after oral administration. It shows gastrointestinal absorption and bioavailability range between 60 and 85% (8). Time to the maximum concentration of drug in serum (*T*_max_) was approximately between 40 to 80 min, and the maximum concentration of drug in serum (*C*_max_) was around 1 mg/L for a dosage of 200 mg. In a comparison between fasting and non-fasting volunteers, it was found that fasting volunteers showed higher *C*_max_ and shorter *T*_max_ than non-fasting volunteers, which means the presence of food interferes with the absorption of ciprofloxacin ^*^.

Ciprofloxacin has low serum binding protein; it shows a mean protein binding of 39% in 0.5, 1, 2, and 5 mg of ciprofloxacin per liter. Ciprofloxacin has great distribution and tissue penetration; accordingly, drug concentration in most tissues and body fluids is higher than in serum (8). Ciprofloxacin can be metabolized in four ways: the primary ways are oxo-ciprofloxacin and sulfo-ciprofloxacin, and two minor ways are ethylene ciprofloxacin and formyl-ciprofloxacin; they are excreted by urine and feces. Unchanged ciprofloxacin was the major molecule appearing in the urine and feces (5).

### Mechanism of action

**Figure 2:**
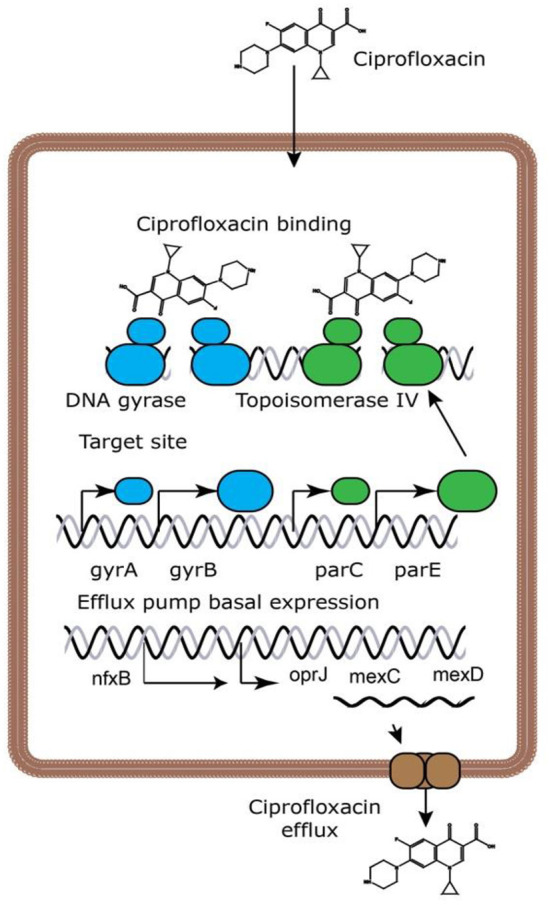
Ciprofloxacin's mechanisms of action (13). Because ciprofloxacin blocks DNA gyrase and topoisomerase IV, DNA replication is slowed and double-stranded DNA breaks are created.

Ciprofloxacin is a broad-spectrum antibiotic that affects its target by inhibiting the DNA gyrase, which is known as topoisomerase II and topoisomerase IV (10). DNA gyrase contains subunits A and B. Quinolones such as ciprofloxacin are believed to prevent subunit A from resealing the DNA double-strand; therefore, single-stranded DNA may result in exonucleolytic degradation (5). In most studies, the effect of ciprofloxacin on DNA gyrase has been emphasized; however, a previous investigation has suggested that ciprofloxacin could affect *Mycobacterium smegmatis* cell wall compounds. It has also been demonstrated that ciprofloxacin, in addition to its effect on DNA gyrase, can cause reduction in the amount of DNA, RNA, and protein, as well as phospholipids, galactose, arabinose, glucosamine, and the mycolic acid of the *M. smegmatis* cell wall. However, these findings should be confirmed in the further studies (Figure 2) (11). Ciprofloxacin affects several Gram-positive bacteria such as *Staphylococcus, Streptococcus, Enterococcus, Bacillus* spp., and *Mycobacterium*. Furthermore, ciprofloxacin shows an acceptable *in vitro* activity against most Gram-negative bacteria strains such as most species of *Enterobacteriaceae, N. gonorrhoeae, Neisseria meningitides, Haemophilus influenza, Moraxella catarrhalis, P. aeruginosa*, and *Legionella* species (5, 12). According to a study, the rank order of *in vitro* activities of seven FQs against 140 clinical *Acinetobacter baumannii* isolates was in the following order: clinafloxacin > gatifloxacin > levofloxacin > trovafloxacin > gemifloxacin = moxifloxacin > ciprofloxacin ^*^. Noteworthy, the inhibitory effects of ciprofloxacin against different Gram-positive and Gram-negative bacteria and a schematic view of this antibiotic's clinical usage are presented in Tables 1, 2, respectively.

**Table 1:** The inhibitory effect of ciprofloxacin against various Gram-positive and Gram-negative bacteria.

**Bacteria**		**MIC of ciprofloxacin**	**References**
Gram-positive bacteria	*Staphylococcus saprophyticus, Staphylococcus epidermidis*, and MRSA	MIC range 0.12–1 mg/L	(5)
	*Streptococcus pneumoniae*	MIC range 0.5–6.3 mg/L	
	*Enterococcus faecalis*	MIC range 0.5–6.3 mg/L	
	MRSA	0.1–0.8 mg/L	(14)
	*Streptococcus pyogenes*	0.5–1.6 mg/L	
	*Enterococcus faecalis*	0.8–25 mg/L	
	MRSA	1 mg/L	(8)
	*S. pneumoniae* and *E. faecalis*	2 mg/L	
	*Streptococcus pyogenes*	4 mg/L	
	*Staphylococcus spp. Enterococcus* spp.	0.5 mg/L	(15)
	*Streptococcus serogroups A*	1 mg/L	
	*MRSA S. pneumoniae*	0.5 mg/L	(16)
	*Enterococcus faecalis*	2 mg/L	
	*Mycobacterium tuberculosis*	MIC range 0.5–1 mg/L	(17)
	*Bacillus anthracis*	0.03 mg/L	(18)
Gram-negative bacteria	*Escherichia coli*	MIC range 0.004–0.25 mg/L	(19)
		≤0.06 mg/L	(16)
		MIC range 0.01–2 μg/ml	(14)
		≤0.25 mg/L	(20)
		8–128 mg/L	(21)
		32–512 mg/L	(22)
	*Klebsiella pneumoniae*	≤0.06–0.125 μg/ml	(16)
		0.03 mg/L	(15)
		0.008–0.12 mg/L	(19)
		0.005–0.1 mg/L	(14)
	*Proteus mirabilis*	MIC <1 mg/L, and only 20% of the strains had MIC ≥1 mg/L	(23)
		MIC range ≤0.06–0.125 mg/L	(16)
		≤1 mg/L	(24)
		≤0.01–0.1 mg/L	(14)
	*Haemophilus influenzae*	0.008–0.015 mg/L	(19)
		≤0.01 mg/L	(14)
		MIC range 0.015–0.03 mg/L	(25)
	*Moraxella catarrhalis*	0.023–0.25 mg/L for β-lactamase-mediated isolates and 0.047–0.125 mg/L for non-β-lactamase-mediated isolates	(26)
		MIC range 0.002–2 mg/L	(27)
		MIC range 0.015–0.06 mg/L	(28)
	*Legionella pneumophila*	≤0.125 mg/L	(29)
		0.06 mg/L	(30)
		0.015–0.03 mg/L	(31)
	*Neisseria meningitidis*	≤0.01 mg/L	(14)
		0.006 mg/L	(32)
	*Neisseria gonorrhoeae*	0.015 mg/L	(33)
		0.008 mg/L	(34)
	*Pseudomonas aeruginosa*	0.5–8 mg/L (the susceptibility of *P. aeruginosa* to ciprofloxacin was 80%)	(35)
		1 mg/L (the percentage of susceptibility of *P. aeruginosa* was 90%)	(25)
		5 mg/L	(36)
		0.016 mg/L	(37)
	*Acinetobacter baumannii*	≤0.03–>128 mg/L	(38)

MRSA, methicillin-resistant S. aureus; MIC, minimum inhibitory concentration.

**Table 2:** Schematic view of clinical usage of ciprofloxacin.

**Infection**	**Notes**	**References**
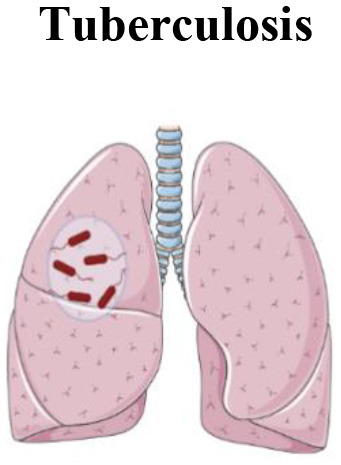	MIC of ciprofloxacin against Mtb ranges between 0.5 and 1 mg/L, and a single oral 750-mg dose of ciprofloxacin has been shown to produce a serum level of 2.01 mg/L, with a bronchial tissue level of 4.86 mg/kg	(39)
	Ciprofloxacin may be effective in treating Mtb, especially in patients with HIV infections and MDR-TB, in combination with other anti-mycobacterial drugs	(40)
	According to WHO consolidated guidelines on tuberculosis, ciprofloxacin is no longer recommended for treating drug-resistant Mtb	(41)
	Mtb sensitivity to ciprofloxacin can be decreased after short exposure courses, which makes this drug ineffective in treating Mtb	(42)
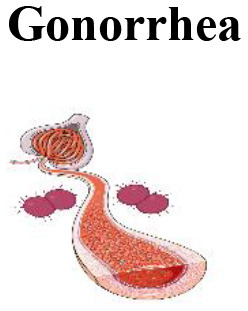	The CDC declared that ciprofloxacin is no longer recommended for the treatment of gonorrhea	(43)
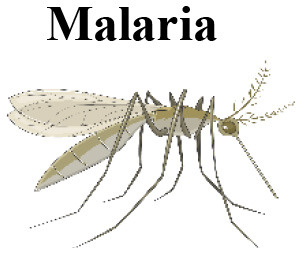	A combination of ciprofloxacin with rapidly acting antimalarial agents such as mefloquine can be a valuable treatment for resistant *Plasmodium falciparum* infections	(44)
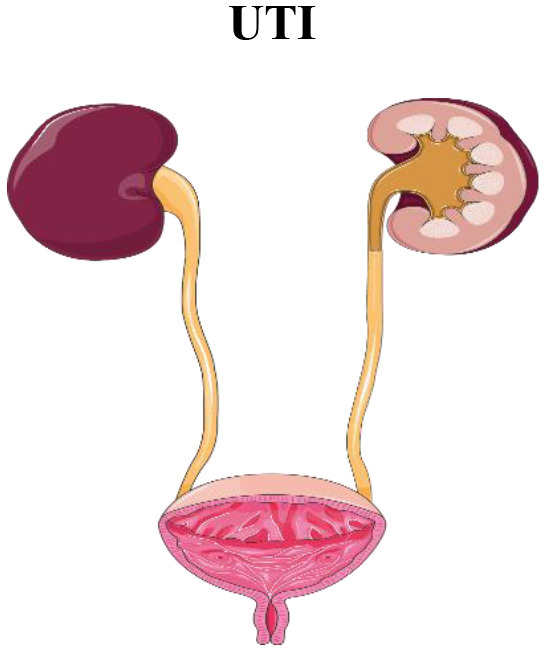	Ciprofloxacin is the most commonly prescribed FQ for the empirical treatment of UTIs because of its availability in oral and intravenous forms	(45, 46)
	Ciprofloxacin is effective for treating acute uncomplicated cystitis in 3-day regimens. However, having a propensity for side effects suggests using ciprofloxacin for more important diseases and considers it an alternative drug for acute cystitis	IDSA guideline (2010 update)
	For treating acute pyelonephritis, an oral 500-mg dose of ciprofloxacin twice a day for seven days with or without an intravenous 400-mg of ciprofloxacin is recommended in regions with <10% of uropathogens resistance	(47)
	Considering the adverse reactions of ciprofloxacin, FDA has recommended not to use ciprofloxacin for uncomplicated UTIs when other choices are available	(48)
	During the last decade, the resistance of uropathogens against ciprofloxacin has increased. In a 10-year follow-up of *E. coli*, a significant increase in ciprofloxacin resistance from 1.8 to 15.9% was observed	(49)
	Ciprofloxacin ER, a once-daily formulation with delayed release, achieves a higher Cmax and has more rapid bacterial killing, which makes it a valuable option for treating out-patient UTIs	(50)
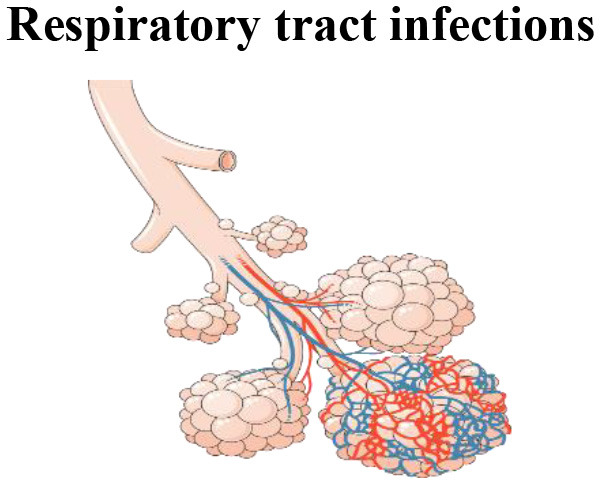	Ciprofloxacin is effective against the most frequent bacterial respiratory pathogens such as *H. influenzae, S. pneumoniae, M. catarrhalis*, and *P. aeruginosa* and can be used to treat complicated and severe lower respiratory tract infections	(51)
	Ciprofloxacin can be used for treating pneumonia (mainly nosocomial), and chronic bronchitis, as well as CF	(51)
	A combination of oral ciprofloxacin with a nebulized antibiotic^*^ (is suggested as first-line therapy), and a 2-week treatment of ciprofloxacin for CF patients who are chronically infected with *P. aeruginosa* is recommended	(52)
	Ciprofloxacin dry powder inhaler was developed for targeted lung delivery, which achieves a high concentration of ciprofloxacin in the lungs with low systemic exposure	(51, 53)
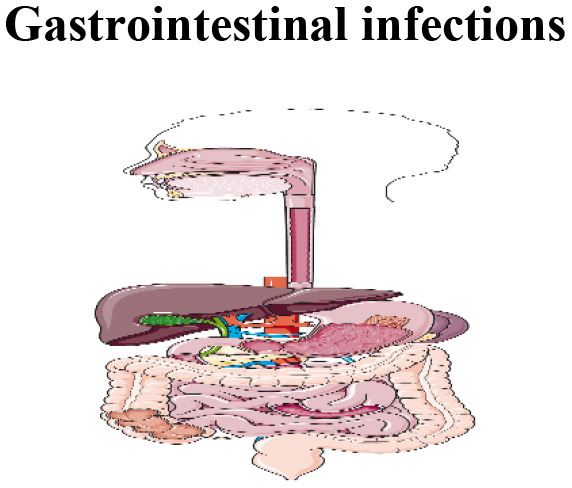	Ciprofloxacin has great mucosal tissue levels and low MICs against *Helicobacter pylori*, but it has failed to eradicate this bacterium because of reduced antibiotic activity in a low pH environment and increased ionization and gastric mucus trapping of ciprofloxacin	(54)
	Ciprofloxacin is the critical choice for treating adult patients with typhoidal and severe non-typhoidal salmonellosis with spreading infection beyond the intestinal tract	(55)
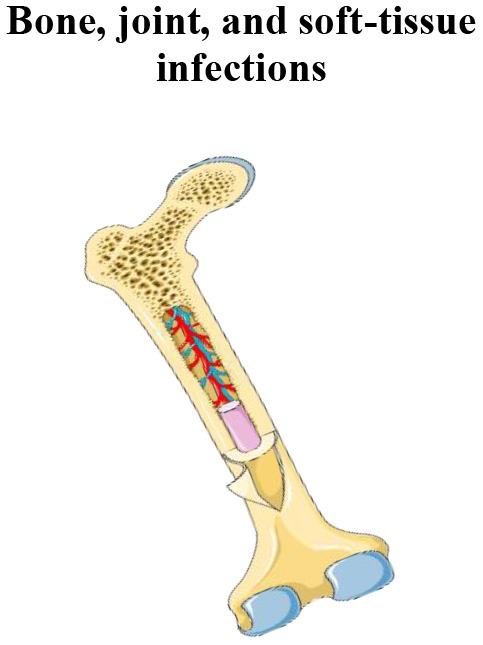	Administration of an oral 750–1,000 mg dose of ciprofloxacin every 12 h can cure most cases of Gram-negative osteomyelitis or mixed infections with *S. aureus* Concerning the increasing rate of resistance against ciprofloxacin, this antibiotic should not be used for the treatment of simple SSTIs but should be reserved for patients with allergies to β-lactams	(56) (57)
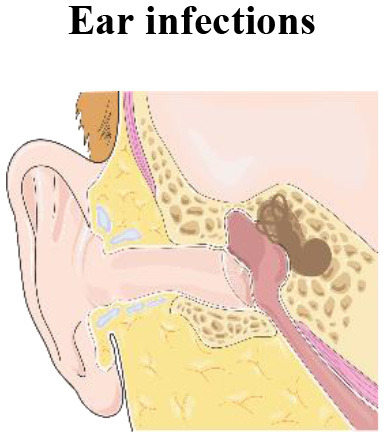	Topical ciprofloxacin has the advantages of direct contact with infected tissue, excellent empiric coverage, non-ototoxicity, and no risk of musculoskeletal complications, which often are associated with systemic use	(58)
	Overuse has increased the prevalence of ciprofloxacin-resistant otologic infections in recent years, which can cause serious challenges in treating ear infections due to the limited options for topical therapy	(59)
	The results of a study indicated that ciprofloxacin was ineffective for treating ciprofloxacin-resistant infections, and other alternatives should be explored	(59)
	The results showed that ciprofloxacin was the most effective antibiotic for the treatment of CSOM, with 93.7% sensitivity of *P. aeruginosa* isolates and high susceptibility rates in Staphylococci, *Klebsiella*, and *Proteus* spp	(60)
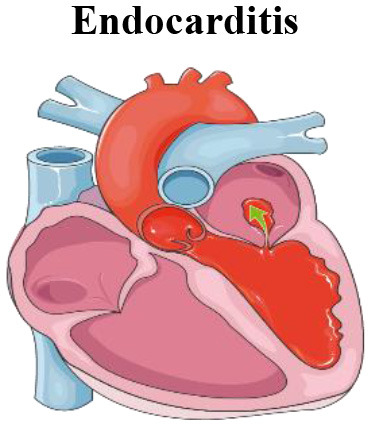	Oral ciprofloxacin is effective against *Pasteurella multocida, Neisseria*, and the HACEK group. It causes endocarditis and can be used in patients with low tolerance to β-lactams A combination of oral ciprofloxacin and rifampicin successfully treats right-sided Staphylococcal endocarditis; however, increasing resistance to these agents is a concern	(61, 62) (63)

CDC, Centers for Disease Control and Prevention; UTI, urinary tract infection; ER, extended-release; FQ, fluoroquinolones; IDSA, Infectious Diseases Society of America; CF, cystic fibrosis; MIC, minimum inhibitory concentrations; SSTIs, skin and soft tissue infections; Mtb, Mycobacterium tuberculosis; CSOM, chronic suppurative otitis media.

* Mainly inhaled colistimethate sodium.

### Anti-biofim effects

Biofilm is made up of cell masses that are located in an environment in their extracellular matrix. This matrix contains polysaccharides, proteins, nucleic acids, and lipids. Biofilms are involved relatively in 80% of human infections (64). Biofilm is one of the most essential factors in developing tolerance against antimicrobial agents (65). Ciprofloxacin is an antibiotic agent that has the potential to control biofilm (66). Reffuveille et al. studied the anti-biofilm effect of ciprofloxacin on *E. coli* and *P. aeruginosa*. The percentage of biofilms remained in the primary biofilms after growth in a medium containing ciprofloxacin (320 ng/ml or 20 μM) + carboxy-TEMPO (4-carboxy-2, 2, 6, and 6- tetramethylpiperidine 1-oxyl) were 0.7 and 13% in *P. aeruginosa* PA14 and *E. coli* O157, respectively. However, using TEMPO without ciprofloxacin revealed that 40% of *P. aeruginosa* and 29% of *E. coli* remain. Hence, it can be concluded that the presence of ciprofloxacin is necessary for decreasing biofilm (67).

Verderosa et al. also evaluated the effect of ciprofloxacin-nitroxide and ciprofloxacin-methoxamine hybrids on *P. aeruginosa* PA14 biofilm at 20- and 40-μM concentrations. When using ciprofloxacin -nitroxide at 20 μM, 80% reduction was observed in the total biofilm; however, half of the biofilm biomass was composed of dead cells, which is suggestive of a 90% reduction in the live cell volume. The use of ciprofloxacin -methoxamine at 40 μg indicated a low reduction in biofilm bio-volume (41%). In addition, 91% of 59% of remaining biomass was composed of dead cells, corresponding to an overall reduction of 95% in live-cell volume, showing a 5% improvement compared to 20 μM. As a result, the higher doses of ciprofloxacin -nitroxide have more potential against *P. aeruginosa* but less capability in removing biofilm, which may be due to the release of cellular adhesive contents (such as DNA) into the environment. Ciprofloxacin-methoxamine reduce biofilm volume by 30% at 20 μM and by 35% at 40 μM concertation, which proves that it has less effect on biofilm than ciprofloxacin-nitroxide (68).

Therefore, recent studies have reported the antibiofilm effect for ciprofloxacin against Gram-negative bacteria. However, these data are limited, and the exact interaction of this antibiotic with bacterial biofilm is not reported. Therefore, future studies should be evaluated molecular and microscopic interactions of ciprofloxacin with the biofilm community of microorganisms; additionally, the anti-biofilm activity of ciprofloxacin should be assessed against multi-species biofilm.

## Resistance mechanisms against ciprofloxacin

Antibiotic resistance is one of the most severe public health issues facing the globe today. Antibiotic-resistant organisms can quickly spread, posing a hazard to populations in the form of novel infectious disease strains that are more difficult to cure and treat (69). Treatment failures may occur due to microbial resistance to effective broad-spectrum antibiotics. Treatment failures and difficult-to-treat infections could lead to a high death rate. Drug target mutations (DNA gyrase and DNA topoisomerase IV), mutations that limit drug accumulation, and plasmids that shield cells from ciprofloxacin's deadly effects are the three mechanisms of ciprofloxacin resistance that have been found (70).

### Alterations in target enzymes

Ciprofloxacin resistance in topoisomerase IV or gyrase can result from a single amino acid change. The amino-terminal domains of *GyrA* (residues 67 to Tyr122 for GyrA, Tyr120 for *ParC*) or *ParC*, which are covalently bound to DNA in an enzyme intermediate (106 for *E. coli* numbering), are where these resistance mutations are most frequently detected (residues 63–102). They are near the tyrosine active site. This domain is referred to as the quinolone resistance determining region (QRDR) of *GyrA* and *ParC* (71).

Quinolone resistance has also been linked to changes in specific domains of *GyrB* and *ParE*; however, these alterations are far less common in resistant clinical bacterial isolates than mutations in *GyrA* or *ParC*. Ciprofloxacin resistance has increased with sequential mutations in both target enzymes. High-level quinolone resistance is frequently associated with mutations in gyrase and topoisomerase IV in several species (72).

### Altered drug permeation

In Gram-positive bacteria, active efflux transporters are the main mechanism for reducing cytoplasmic drug concentrations. It has not been demonstrated that decreased diffusion through the cytoplasmic membrane is a form of resistance. Reduced outer membrane porin diffusion channels, which are necessary for ciprofloxacin to enter the periplasm, may be a factor in the development of resistance in Gram-negative bacteria and cooperate with basal or elevated expression of efflux transporters (72).

Porins are the main route for hydrophilic antibiotics like FQs to enter the bacterial outer membrane. Coexisting resistance mechanisms such as efflux pumps or antibiotic degrading enzymes are amplified by lower antibiotic uptake due to alterations in porin expression, resulting in high-level resistance (73).

### Plasmid-mediated quinolone resistance

Horizontal transference has been identified as the principal method for spreading quinolone resistance globally since 1998, when the primary plasmid-mediated quinolone resistance gene (PMQR) was first identified in a *K. pneumoniae* strain in the USA (74). The lowest inhibitory concentrations (MIC) of FQs, which typically prevent their *in vitro* detection, impart a modest growth in the presence of these resistance determinants. Furthermore, taking into account high-degree resistance to widen, PMQR might contribute to an increase in the occurrence of spontaneous mutations in QRDRs (72, 74, 75).

Inducing low susceptibility to these drugs by protecting the binding site in DNA-gyrase (*qnr* gene), modifying the drug enzymatically (*aac(6')-Ib-cr* gene), and expelling the agent from its site of action by coding for efflux pumps (*oqxAB* and *qepA* genes) are currently the three main mechanisms of resistance to quinolones related to PMQR that are recognized (74, 76).

**Figure 3:**
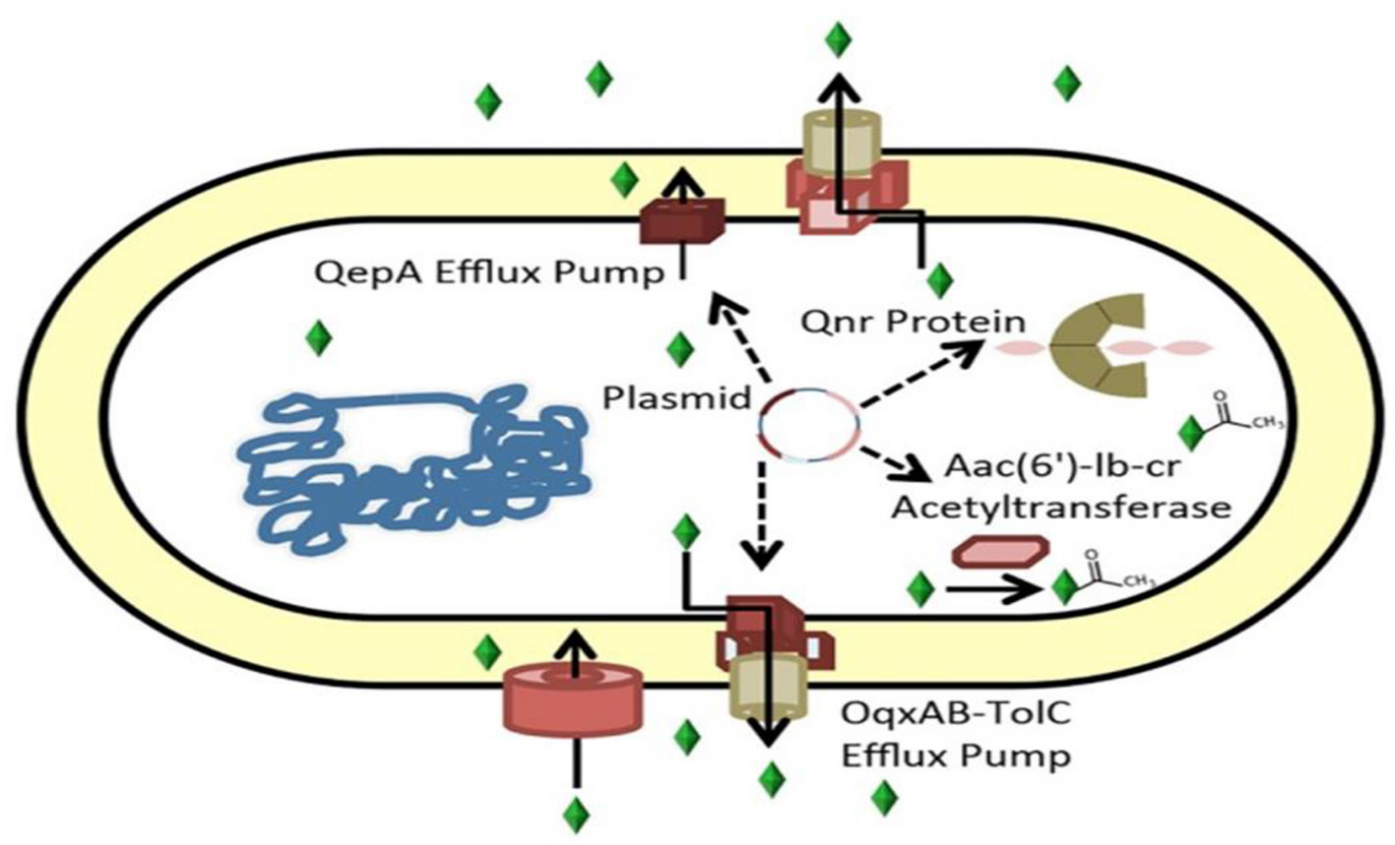
Mechanisms of ciprofloxacin resistance carried by plasmids (82). Genes encoding the ciprofloxacin efflux pumps can be found in plasmids. Aac(6')-Ib-cr, an aminoglycoside-modifying acetyltransferase that acetylates and inactivates ciprofloxacin, or QepA or OqxAB, the Qnr protein, which binds gyrase.

PMQR genes consist of six *qnr* genes (*qnrA, qnrB, qnrC, qnrD, qnrS*, and *qnrVC*) encoding gyrase-protection repetitive peptides *oqxAB, qepA*, and *qaqBIII* encoding efflux pumps (77, 78); and *aac(6′)-Ib-cr* encoding an aminoglycoside and quinolone inactivating acetyl-transferase (79). These genes can synergize with chromosomal *gyrA* and *parA* mutations, increase the mutant prevention concentration of quinolones, interfere with quinolone action in apparently susceptible bacteria harboring them (80), and confer evolutionary fitness unrelated to quinolone resistance (Figure 3) (81).

In some *Enterobacteriaceae* species, the co-existence of mutations in the QRDR and PMQR genes can may occur. Additionally, QRDR mutations that increase FQs resistance can be encouraged by the presence of PMQR determinants (83). According to the findings of the Egyptian study, a high level of resistance to FQs is conferred by the accumulation of PMQR genes and QRDR mutations (84).

## Mechanism of resistance in gram-negative bacteria

### Neisseria

Single nucleotide polymorphisms (SNPs) in *gyrA* alone confer low- to intermediate-level resistance in *N. gonorrhea*, whereas high-level resistance necessitates one or more specific concurrent mutations in *parC*. These changes can be easily selected and transferred to other gonococci by exposing them to sub-inhibitory ciprofloxacin doses (85).

A missense mutation in *gyrA* (S91F) within the QRDR has been demonstrated to cause a 100-fold increase in ciprofloxacin resistance. A subsequent mutation at codon 95 (D95N) resulted in a two-fold increase in ciprofloxacin resistance. Higher levels of quinolone resistance required mutations in *parC* in addition to those in *gyrA*. These *parC* mutations were found in codons 88 (S88P) and 91 (E91K) of the *parC* gene (85). Additional GyrA/ParC amino acid change patterns were later discovered in ciprofloxacin-resistant bacteria worldwide (86). Ciprofloxacin resistance in Gonorrhea appears unaffected by mutations in the *gyrB* and *parE* genes (86).

As mentioned, the most common combinations of amino acid substitutions in the GyrA and ParC proteins conditioning resistance to FQs are S91F + D95G/A in GyrA and S87R in ParC (87). This combination was found in more than 40% of *N. gonorrhoeae* strains resistant to FQs and conditioned the MIC of ciprofloxacin from 4 to 32 mg/L (87). The frequency of individual mutations in the *gyrA* and *parC* genes varies (88). A mechanism increasing FQ MIC values, based on the overproduction of NorM membrane pump proteins, was also described in single *N. gonorrhoeae* strains (86–89).

In gonococci, four efflux pump systems (MtrCDE, MacAB, NorM, and FarAB) have been discovered in most strains. The MtrCDE, MacAB, NorM, and FarAB systems belong to the RND, ABC, MATE, and MF families, respectively, which have been proven to identify antimicrobials previously or currently approved for gonorrhea treatment (89, 90).

The MICs for ciprofloxacin-resistant *N. meningitides* isolates have been reported to range between 0.06 and 0.25 g/ml, with mutations in the QRDR of the gyrase-encoding gene *gyrA* being responsible for the majority of this resistance (91–93). According to a Chinese study, mutations in the QRDR of *gyrA* related with quinolone resistance substitutions were observed in all of the 51 ciprofloxacin-non susceptible *N. meningitides* strains, of which 49 strains harbored the typical substitution of threonine to isoleucine at amino acid position 91 (T91I). The other two ciprofloxacin-intermediate strains, harbored aspartate to asparagine substitutions at amino acid position 95 (D95N). No additional mutations were observed in the QRDRs of *gyrB, parC*, or *parE*. Furthermore, sixteen *gyrA* alleles (R1–R16), were defined in 51 ciprofloxacin-non susceptible isolates. Most of ciprofloxacin resistance-conferring alleles were transmitted through horizontal gene transfer (93).

The *gyrA* gene of *N. meningitidis* is 95% identical to the *gyrA* gene of *N. gonorrhoeae*. Mutations in the *gyrA* gene have been associated with ciprofloxacin resistance in *N. meningitidis*. The QRDR from the resistant *N. meningitidis* contained a mutation that resulted in an Asp95-to-Asn change. This known change in the *N. gonorrhoeae gyrA* gene's QRDR raises ciprofloxacin MICs to levels similar to those seen in this strain (94).

In the QRDR of *gyrA*, nearly all previously identified ciprofloxacin-resistant *N. meningitidis* isolates had Ile (I) or Phe (F) mutations at position 91 (92, 95–97). In *N. meningitidis*, further mutations in *gyrA* (D95N and T193A), as well as *parC* (D86N, S87R, and E91G), have been linked to increased ciprofloxacin MICs (92, 93, 97, 98). Chen et al. found that all quinolone-resistant *N. meningitidis* isolates contained mutations in T91 and/or D95 of GyrA, with seven isolates also possessing ParC mutations and displaying higher MICs. The specific *Neisseria lactamica* donors of seven mutation-carrying *gyrA* alleles (*gyrA*92, *gyrA*97, *gyrA*98, *gyrA*114, *gyrA*116, *gyrA*151, and *gyrA*230) and the *Neisseria subflava* donor isolate of *gyrA*171 were discovered by genomic analysis. Transformation of *gyrA* fragments from these donor strains into a meningococcal isolate raised its ciprofloxacin MIC from 0.004 g/ml to 0.125 or 0.19 g/ml and to 0.5 g/ml with the further transformation of an additional ParC mutation, according to their findings. Over fifty percent of quinolone-resistant *N. meningitides* strains acquired resistance through horizontal gene transfer from three commensal *Neisseria* species (97).

According to a study conducted in Brazil, all of the ciprofloxacin -resistant *N. meningitides* isolates possessed a Thr to Ile mutation at the QRDR of the *gyrA* gene's amino acid 91. No further mutations were identified in the *gyrA* or *parC* QRDRs (91). According to a study in Spain, single mutations in the *gyrA* (with Thr-91 to Ile being the most common substitution found) of ciprofloxacin-resistant *N. meningitidis* were the primary mechanism implicated. Four distinct *gyrA* substitutions were found in two meningococci. There were no changes in the *parC* and *gyrB* genes' QRDRs. However, three strains had a His-495 to Asn substitution in the *parE* gene. In addition, two distinct mutations in the *mtrR* gene that impact the expression of the MtrCDE efflux mechanism were discovered (99).

### Pseudomonas aeruginosa

Two basic pathways of ciprofloxacin resistance in *P. aeruginosa* have been thoroughly explored. Major contributors to ciprofloxacin resistance in *P. aeruginosa* are mutations in the ciprofloxacin target-encoding genes *gyrAB* and *parCE* that decrease the affinity of DNA gyrase or topoisomerase for ciprofloxacin (13). Furthermore, overexpression of efflux pumps to lower antibiotic intracellular concentrations promotes ciprofloxacin expulsion from *P. aeruginosa* cells due to mutations in efflux pump regulatory genes. It has become apparent that a wide number of additional genes can play a role in ciprofloxacin resistance and that resistance evolves through a mix of alleles, underscoring the multifactorial character of the ciprofloxacin resistance development process (13). Bacteria with both target-site mutations and efflux overexpression were more resistant to ciprofloxacin than bacteria with only individual mutations (100).

Sequence variants in which the Thr at position 83 in GyrA is replaced by Ile and the Ser at position 87 in ParC is replaced by a Leu are the most commonly occurring alterations associated with ciprofloxacin resistance in *P. aeruginosa* isolates from patients and *in vitro* evolved isolates (100).

The second most common GyrA variation occurs at position 87, where Asn, Tyr, or Gly residues replace aspartate (13). The presence of alternative amino acid residues at these positions decreases gyrase's affinity for ciprofloxacin, providing a molecular explanation for the GyrA variations' increased ciprofloxacin resistance (101). GyrA and ParC variants are more common than GyrB and ParE variants, possibly because alterations in GyrB, and ParE sequences give lower-level ciprofloxacin resistance. In clinical isolates of *P*. *aeruginosa*, resistance alleles in both *gyrA* and *parC* give stronger ciprofloxacin resistance than resistance alleles in only *gyrA* (102–104).

Four efflux pumps in *P. aeruginosa* are known to efflux FQs: MexCD-OprJ, MexEF-OprN, MexAB-OprM, and MexXY-OprM (105–107). System-specific regulatory proteins regulate efflux pump gene expression, and mutations in these regulators cause efflux pump overexpression (108, 109). Two efflux pumps are overexpressed. Most typically, MexCD-OprJ and MexEF-oprN have been implicated with ciprofloxacin resistance. Overexpression of MexCD-OprJ occurs in *P. aeruginosa* isolates from Cystic Fibrosis (CF) and non-CF patients and is caused by mutations in the *nfxB* gene (110, 111).

Overexpression of MexEF-OprN occurs in isolates of *P. aeruginosa* from CF and non-CF patients due to mutations in the *mexS* gene, which result in overexpression of MexT. The MexEFoprN genes are regulated by the transcription factor MexT (106).

Furthermore, overexpression of MexXY-OprM in clinical isolates of *P. aeruginosa* has been demonstrated to confer ciprofloxacin resistance at lower levels. Mutations in the regulator gene *mexZ* have been blamed for most MexXY-OprM overexpression (100). Experiments demonstrate that a *gyrA*-resistant allele mutation is required for ciprofloxacin resistance, with other mutations enhancing resistance further (112). GyrA's great affinity for ciprofloxacin makes it possible for bacteria to harbor mutations in the regulatory genes of efflux pumps, yet a wild-type *gyrA* allele may still be vulnerable to the drug (100).

### Campylobacter

The most prevalent mechanism of ciprofloxacin resistance in *Campylobacter* is a single point mutation C257T in the *gyrA* gene, located within the QRDR resistance (113). This causes a Thr to Ile amino acid change in the Gyrase A subunit at position 86 (114). Other mutations in the *gryA* gene have been linked to increased ciprofloxacin resistance but at lower doses and frequencies (115–117). In *Campylobacter* spp., polymorphisms in the *gyrB* gene have been ruled out as a cause of quinolone resistance. The *gyrA* mutation interacts with the most frequent *Campylobacter* drug efflux pump, CmeABC, to promote the development of ciprofloxacin-resistant bacteria when its expression is raised (116, 117). Overexpression of the CmeABC efflux pump does not result in ciprofloxacin resistance without the *gyrA* gene mutation (114, 117, 118).

The 16-bp inverted repeat (IR) in the *cmeR-cmeABC* intergenic region is one more element that heightens resistance. The percentage of resistant isolates increases and the average ciprofloxacin MIC increases when this mutation coexists with the *C257T-gyrA* mutation. Recently found and spreading, *RE-cmeABC* is a variant of the *cmeABC* gene that increases ciprofloxacin resistance (118). Ciprofloxacin resistance may be indirectly impacted by changes in other genes. Variations in the mutant frequency decline gene (*mfd*), for instance, may be involved because silencing of this gene has been shown to 100-fold reduce mutation rates (117).

### Haemophilus influenzae

Ciprofloxacin resistance in *H. influenzae* is associated to chromosome-mediated mutations in the QRDRs of the genes producing DNA gyrase and topoisomerase IV, including *gyrA, gyrB, parC*, and *parE*. *GyrA* (at Ser84 and Asp88) and *parC* (at Gly82, Ser84, and Glu88) had more amino acid changes than *gyrB* and *parE* (119). Puig et al. found that strains with a single alteration in GyrA or one change in GyrA plus one in ParC had ciprofloxacin MICs of 0.12 to 2 g/ml.

In contrast, those with three or four changes (in GyrA, ParC, and ParE) had higher MICs (8–16 g/ml) (120). Ser84 to Leu or Tyr and Asp88 to Tyr, Asn, or Gly were the most common alterations in GyrA, which have been linked to resistance in *H. influenza* (119). In ParC, the most common changes were Ser84Ile and Glu88Lys (119) and Ser84Arg (119).

### Enterobacteriaceae

Ciprofloxacin resistance in *Enterobacteriaceae* has been extensively researched (121, 122). The accumulation of mutations in the genes encoding the two quinolone targets: DNA gyrase and topoisomerase IV in *E. coli* is a major contributor to resistance and decreased sensitivity to quinolones (123).

It may just take one change to the *E. coli* gene *gyrA* to result in large levels of nalidixic acid resistance. However, additional, progressive mutations in the topoisomerase IV or *gyrA* genes are necessary for high-level FQs resistance, including ciprofloxacin. *Escherichia coli* was the only species in a study of eight *Enterobacteriaceae* species where multiple mutations in *gyrA* were necessary for high-level FQ resistance. The most frequent *gyrA* mutations identified in clinical, veterinary, and laboratory strains of *E. coli* occur at codon 83. This Ser residue is most frequently changed to Leu in *E. coli* isolates with high levels of nalidixic acid resistance and lower susceptibility to FQs^*^.

Strains with a somewhat higher resistance to FQs had an extra mutation, most frequently at codon Asp87. The high occurrence of mutations at Ser83, however, has a plausible explanation because strains with a single mutation at Ser83 were considerably more resistant to FQs than those with a single mutation at Asp87^*^.

Increased drug extrusion caused by overexpression of AcrAB-TolC, the principal efflux pump reported in *Enterobacteriaceae*, on the other hand, is a major source of worry because it confers cross-resistance to a variety of unrelated chemicals, including antimicrobials. Other efflux systems, such as AcrEF and EmrAB, have been reported to engage in the extrusion of antimicrobial compounds to a lesser amount (124).

Increased efflux has been identified as the main mechanism for the development of quinolone resistance in *Salmonella*. On the other hand, in these bacteria, decreased OmpF porin synthesis has occasionally been linked to the MDR phenotype. Furthermore, according to a study, the ParC T57S substitution was common in strains exhibiting the lowest MICs of ciprofloxacin, while increased MICs depended on the type of GyrA mutation. PMQR genes represented a route for resistance development without target-site mutations (125).

According to Azargun et al., high-level ciprofloxacin resistance in *Enterobacteriaceae* is linked to DNA gyrase and topoisomerase IV mutations as a primary mechanism and PMQR genes *acrB* efflux pump gene expression, and outer membrane *ompF* gene expression. Ciprofloxacin resistance is increased due to twin mutations in *gyrA* and *parC* (124). PMQR genes are not the critical mechanism of ciprofloxacin resistance in uropathogenic *E.coli* in South Iran, according to Malekzadegan et al. (126).

### Legionella pneumophila

*Legionella pneumophila* resistance to ciprofloxacin is most typically linked to changes in *gyrA, gyrB, parC*, and *parE* genes. Mutations affecting codons 83 and 87 of the *gyrA* QRDR have been linked to the *in vitro* selection of *L. pneumophila* strains with high-level ciprofloxacin resistance (127). However, mutations in *gyrB* and *parC* have also been identified (127). *In vivo*, only mutations at codon 83 of the *gyrA* gene have been described (128, 129).

Using next-generation DNA sequencing (NGS), Shadoud et al. demonstrated that the 248CT (T83I) mutation-carrying L. pneumophila mutant population was rapidly selected *in vivo* in two legionellosis patients treated with ciprofloxacin, increasing from 1.05% of the total *L. pneumophila* lung population at the time of diagnosis to 94% after a few days of FQ treatment (129).

### Moraxella catarrhalis

According to a study, an amino acid substitution of Thr80 to Ile in GyrA causes *M. catarrhalis* to have low-level resistance to FQs (130). FQ targets gyr and par were also sequenced in another work on isolates with decreased FQ resistance that were produced by stepwise selection in levofloxacin. GyrA (D84Y, T594dup, and A722dup), GyrB (E479K and D439N), and ParE (Q395R) were shown to have six new mutations that contribute to *M. catarrhalis* resistance to FQs (131). According to a Polish study, *M. catarrhalis* FQ resistance is linked to amino acid changes in the *gyrA* and *gyrB* genes. G412C and four silent transition mutations were found in the *gyrA* gene. Two identical silent mutations and the substitution A1481G occurred in the *gyrB* gene (132).

### Acinetobacter baumannii

Resistance to ciprofloxacin in *Acinetobacter baumannii* is advanced *via* unique techniques, one of which is the modifications that took place within the expression of the efflux pumps. The efflux pump in *A. baumannii* is the AdeABC pump and is of great significance in phrases of resistance advent (133).

This efflux pump has a three-part structure and is a member of the resistance-nodulation-cell department (RND) family: AdeB is a multidrug transporter, AdeC is an outer membrane protein, and AdeA is a membrane fusion protein. AdeS is a sensor kinase, while AdeR is a response regulator. Together, they form the -component system (AdeR-AdeS) that tightly controls the adeABC operon. Both point mutations in AdeRS and the insertion sequence (IS) Aba-1 insertion upstream of the adeABC operon have been implicated in the overexpression of the AdeABC efflux pump^*^.

The presence of quinolone resistance (*qnr*) genes on the plasmid, which results in a low-level resistance to quinolones, is another mechanism that results in resistance to ciprofloxacin (134). A mutation in the quinolone resistance-determining regions (QRDR), which affects the target enzymes of DNA gyrase (*gyrA*) and topoisomerase IV (*parC*), is another important mechanism. The main effects of quinolones are on target enzymes like DNA gyrase, which block the transcription process by attaching to and mutating this enzyme's gene (135).

Sequencing results in several studies revealed a serine to leucine mutation at position 83 of the *gyrA* subunit, indicating that Ser83Leu substitution is the primary mutation in *A. baumannii* for FQ resistance (136). In ciprofloxacin-resistant isolates from a different investigation, Ala84Pro or Gly81Val mutations in the *gyrA* gene were found. Quinolones' target in *A. baumannii* is topoisomerase IV, and mutations at *parC* residues Ser80 and Glu84 contribute to decreased fluoroquinolone sensitivity^*^.

Two clinical isolates from another study had mutations in *parC* without *gyrA*, suggesting that *parC* might not only be a secondary goal for quinolones but is as critical as *gyrA* to purpose a decreased susceptibility to FQs in *A. baumannii* (136). ParC mutations are typically in conjunction with mutations in *gyrA* and are needed to gather a high degree resistance to quinolones. *Acinetobacter baumannii* can resist FQs with just a single point mutation in DNA gyrase, but concurrent mutations in the QRDR regions of the *gyrA* and *parC* genes are projected to significantly contribute to high-degree FQs resistance (136).

A study found that the Serine 83 to Leucine mutation was present in the DNA gyrase subunit A's QRDR in isolates that were resistant to ciprofloxacin (GyrA). Furthermore, among isolates that were resistant to ciprofloxacin, researchers were unable to detect ParC mutations or plasmid-mediated quinolone resistance (qnrA). They came to the conclusion that a mutation in GyrA, with the presence of efflux pumps serving as a secondary motive, is the primary source of ciprofloxacin resistance in *A. baumannii* isolates from burn infections (137).

According other study in Iran, the prevalence rates of *qnrA, qnrB, qnrS, AdeA, AdeB*, and *AdeC* genes among *A*. *baumannii* isolates have been 0, 0, 3.9, 100, 100, and 100%, respectively. In all of the resistant isolates, mutation within the *gyrA* gene became discovered, however, no mutation became visible within the *parC* gene (138).

## Mechanism of resistance in gram-positive bacteria

### Enterococci

In *Enterococci*, ciprofloxacin resistance is mostly caused by chromosomal mutations in the genes encoding quinolone targets, DNA gyrase, and topoisomerase IV, which are mostly found in the QRDR (139). Resistance-associated mutations have been discovered in the *gyrA* gene (Ser83Arg, Ile, or Asn; Glu87Lys, Gly) and the parC gene in *E. faecalis* (Ser80Arg, or Ile; Glu84Ala). In *Enterococcus faecium*, mutations in the *gyrA* gene (Ser83Ala, Leu, Ile, Tyr, or Arg; Glu87Leu, Gly, or Lys) and the *parC* gene (Ser83Ala, Leu, Ile, Tyr, or Arg) have been identified (140).

Another well-known mechanism of quinolone resistance is antibiotic externalization *via* efflux pumps. NorA is described in *E. faecium* and EmeA in *E. faecalis* (141). A third resistance mechanism reported in *E. faecalis* is qnr, a protein with a series of pentapeptide repeats identical to the plasmid-borne quinolone resistance genes identified in *Enterobacteriaceae*. This protein protects DNA gyrase by preventing ciprofloxacin from binding to DNA and forming an antibiotic–gyrase complex (142).

### Staphylococcus aureus

It has been well established over the past few decades that the pathogen's capacity for resistance to antimicrobial drugs, particularly methicillin, may contribute to its persistence in the hospital and community (143). Methicillin-resistant *S. aureus* (MRSA) are a global health concern due to their growing resistance to macrolide, lincosamide, and streptogramin B treatments (144). In clinical isolates of *S. aureus*, resistance to ciprofloxacin is caused by both mutations in topoisomerases that impair drug binding effectiveness and increased production of endogenous efflux pumps (72). Amino acid substitutions in residues that make up the drug-binding site, also known as the quinolone resistance-determining area, are the most common types of mutations (72). ParC is the topoisomerase with the highest sensitivity in *Staphylococci* and is thus the major target. The secondary target is DNA gyrase, which is less sensitive. FQs are highly effective for *Staphylococci*; thus, alterations in both enzymes are required to build a resistance that exceeds the MIC breakpoint. A single amino acid substitution will often increase the MIC by 8–16 times (145, 146).

Clinical isolates with strong ciprofloxacin resistance frequently overexpress chromosomally encoded efflux pumps. NorA is responsible for the ciprofloxacin and norfloxacin resistant, while NorB and NorC are responsible for the sparfloxacin and moxifloxacin resistant. Therefore, the overexpression of an efflux pump (NorA) leads to the ciprofloxacin resistance in *S*. *aureus* (72).

After challenging 222 isolates of *S. aureus* with the antibiotic ciprofloxacin, Papkou et al. (147) discovered that a single efflux pump, *norA*, causes widespread variation in evaluability across isolates, and that chemical inhibition of NorA effectively prevents resistance evolution in all isolates. The frequency of efflux pump genes driving ciprofloxacin and antiseptic resistance in MRSA isolates was studied in a study conducted in Iran. According to their findings, the *mdeA* and *qacA/B* genes were detected with the highest (61.7%) and lowest (3.3%) frequency, respectively, among ciprofloxacin-resistant isolates (148).

### Mycobacterium tuberculosis

The second-leading cause of death worldwide among infectious diseases is TB, an old infectious disease caused by *M. tuberculosis* and other species that are closely related to it. Each year, an estimated two–three million people die from TB and its associated complications worldwide (149). DNA gyrase mutations, drug efflux pumps, bacterial cell wall thickness, and pentapeptide proteins (MfpA)-mediated gyrase regulation in *M. tuberculosis* are the ciprofloxacin -resistant mechanism in Mtb (150). Because mycobacteria lack topoisomerase IV, ciprofloxacin resistance mutations are found in the genes encoding gyrase, most commonly in the QRDR of *gyrA*, but sometimes in the QRDR of *gyrB*. Mutations mainly cause FQ resistance in tuberculosis in the *gyrA* gene, the most prevalent at locations 90, 91, and 94, which are associated with high-level resistance (151).

The most prevalent mutations in *gyrA* are found in the QRDR codons 88–94, particularly codons 88, 90, 91, and 94. FQ resistance in *gyrB* is often linked to mutations in codons 500 and 538 (152).

The incidence of *gyrA* mutations does, however, vary geographically. A study's mutational examination of samples from pulmonary TB patients revealed that the majority of the mutations change codons 94 (changing Asp with Gly, D94G), and 90 (replacing Ala with Val A90V). In MDR and treatment failure instances, the D94G mutation was most frequently linked to resistance to FQs. However, many A90V mutations were discovered in recently diagnosed patients (153).

The Mmr efflux transporter is the only efflux pump from the small multidrug resistance (SMR) family in the Mtb genome. It has been linked to *M. tuberculosis* resistance to dyes and antibiotics such as FQs (154). In a systematic assessment of *gyr* mutations, 64% of FQ-resistant *M. tuberculosis* isolates contained mutations in the QRDR of *gyrA*. In 534 resistant isolates, the QRDR of *gyrB* was sequenced, but only 3% exhibited mutations. Eighty-one percent of the *gyrA* mutations were found inside the QRDR, whereas 19% were found outside. In 54% of FQ resistant isolates, mutations in *gyrA* codons 90, 91, and 94 were found (substitutions at amino acid 94 accounted for 37%). Only 44% of the *gyrB* mutations were found inside the QRDR (155). Two amino acid positions, 74 and 88, are related to less prevalent genetic variants in *gyrA* (156).

Multiple mutations and codons 94, 90, and 88 of *gyrA* provided high-level FQ resistance (157). The considerably less common *gyrB* mutations (up to 10%−15%) were generally, but not consistently, associated with lower levels of FQ resistance (155). Nevertheless, combined *gyrA* and *gyrB* mutations could result in a substantially higher resistance level (155, 158). Other efflux pumps that may be involved in FQ resistance include antiporters LfrA and Tap, in addition to the mycobacterial pentapeptide MfpA and the ATPase complex Rv2686c-Rv2687c-Rv2688c operon (159). According to a study, ciprofloxacin-resistant clinical isolates of *M. tuberculosis* had significant efflux pump pstB transcripts in a few isolates, implying that the pump plays a role in resistance (160).

It should be noted that all of the mentioned resistance mechanisms are summarized in Table 3.

**Table 3:** Mechanisms of ciprofloxacin resistance in different bacteria.

**Bacteria**	**Mechanism of resistance**	**References**
*Neisseria gonorrhea*	1. Target-site modification (gyrA SNPs: S91F, D95N, and D95G, in the QRDR and parC SNPs: D86N, S88P, and E91K, in the QRDR) 2. An overexpressed NorM efflux pump	(85, 86, 89)
*Neisseria meningitidis*	1. Mutations in the QRDR of the gyrase-encoding gene gyrA [Ile (I) or Phe (F) mutations at position 91] ✓ Further mutations in *gyrA* (D95N and T193A) and *parC* (D86N, S87R, and E91G) have been linked to increased ciprofloxacin MIC 2. The Over expression of the MtrCDE efflux mechanism (by two distinct mutations in the mtrR gene)	(92, 93, 95, 97–99)
*Pseudomonas aeruginosa*	1. Target-site modification (Most common: replacement of Thr at position 83 in GyrA is by Ile and the Ser at position 87 in ParC by a Leu) 2. Efflux overexpression (MexCD-OprJ, MexEF-OprN, MexAB-OprM, and MexXY-OprM)	(13, 100, 105, 107)
*Campylobacter jejune*	1. Single point mutation C257T in the gyrA gene 2. Overexpression of efflux pump CmeABC 3. Inverted repeat (IR) in the cmeR–cmeABC intergenic region	(113, 114, 118)
*Haemophilus influenza*	1. Amino acid changes in the QRDR of the topoisomerase II and I genes ✓ gyrA (Ser84 and Asp88) and parC (Gly82, Ser84, and Glu88) had more amino acid changes than gyrB and pare	(119)
*Escherichia coli*	1. Mutations in the DNA gyrase (gyrA and gyrB) and topoisomerase IV (parC and parE) are a major contributor to resistance [*gyrA* mutations (Nucleotide substitutions at codon 83)] and additional mutation, most commonly at codon Asp87 2. Overexpression of AcrAB-TolC, (the principal efflux pump)and AcrEF and EmrAB 3. Decreased expression of OmpF	(123, 124)
*Salmonella*	1. Increased efflux (a primary mechanism) 2. Decreased production of the OmpF porin 3. Mutations in gyrA and parC	(125, 126)
*Legionella*	1. Mutation in the gyrA/gyrB and parC/paeE (mostly mutations affecting codons 83 and 87 of the gyrA QRDR)	(127)
*Moraxella catarrhalis*	Amino acid substitutions in gyrA and gyrB gene (Amino acid substitution of Thr80 to Ile in GyrA: low-level resistance)	(130)
*Acinetobacter*	1. Expression of the efflux pumps. (AdeABC pump) 2. Presence of quinolone resistance (*qnr*) genes located on the plasmid, (low-level resistance) 3. Mutation in quinolone resistance-determining regions (QRDR), where the target enzymes of DNA gyrase (*gyrA*) and Topoisomerase IV (*parC*)	(133) (138) (135)
*Enterococci*	1. Chromosomal mutations in gyrA and parC ✓ Resistance-associated mutations have been discovered in the *gyrA* gene (Ser83Arg, Ile, or Asn; Glu87Lys, Gly) and the parC gene in *E. faecalis* (Ser80Arg, or Ile; Glu84Ala) ✓ In *E. faecium*, mutations in the gyrA gene (Ser83Ala, Leu, Ile, Tyr, or Arg; Glu87Leu, Gly, or Lys) and the parC gene (Ser83Ala, Leu, Ile, Tyr, or Arg) have been identified 2. Overexpression of active efflux (NorA in *E. faecium* and EmeA in *E. faecalis*) 3. Target protection (Qnr-like determinants), Binds gyrase, described in *E. faecalis*	(139, 140, 142)
*Staphylococcus aureus*	1. Mutations in the QRDR of DNA gyrase and topoisomerase IV ✓ ParC is major target. The secondary target is DNA gyrase, which is less sensitive 2. Overexpression of the efflux pump NorA	(145, 147)
*Mycobacterium tuberculosis*	1. Mutations in the genes encoding gyrase, most commonly in the QRDR of gyrA, but sometimes in the QRDR of gyrB ✓ The most prevalent mutations in gyrA are found in the QRDR codons 88–94, particularly codons 88, 90, 91, and 94 ✓ FQ resistance in gyrB is often linked to mutations in codons 500 and 538 2. Overexpression of The Mmr efflux pump and other efflux pumps include antiporters LfrA and Tap, in addition to the mycobacterial pentapeptide MfpA and the ATPase complex Rv2686c-Rv2687c-Rv2688c operon	(152, 154, 159)

CIP, ciprofloxacin; QRDR, quinolone resistance determining region; MIC, minimum inhibitory concentration.

## The combine use of ciprofloxacin with different antibacterial agents

### Synergism of ciprofloxacin with aminoglycoside

Synergism of ciprofloxacin and amikacin for *P. aeruginosa* is as follows: total synergism–ciprofloxacin ¼ MIC + amikacin ¼ MIC, partite synergism–ciprofloxacin ½ MIC + amikacin ^1^/_16_ MIC or ciprofloxacin 1/_16_ MIC + amikacin ½ MIC. Time-kill assay affirmed the synergistic activity of ciprofloxacin and amikacin, apparent at as early as 4 h and kept up after that^*^. Combining gentamicin with ciprofloxacin against *E. coli* and *P. aeruginosa* displayed the ideal treatment alternative. However, more *in vivo* and clinical trials are needed to determine the potential treatment regimen based on the combination of these two antibiotics (161).

Additionally, unlike when each antibiotic was used alone, combining tobramycin with either azithromycin or ciprofloxacin enhanced the killing of planktonic *K. pneumoniae* cells and accelerated bacterial clearance in a mouse model of cutaneous abscess infection. Additionally, combining ciprofloxacin and tobramycin increased the bactericidal activity against cells linked to biofilms. In this regard, the antibiotic combinations reduced the number of bacteria from 108 to fewer than 10 colony forming units (CFU) ml^−1^; however, when each antibiotic was used alone, only 500 CFU ml^−1^ of bacteria were recovered (162).

In view of these findings, ciprofloxacin and tobramycin may be used in combination to treat both acute and persistent *K. pneumoniae* infections. The use of the aforementioned combination therapy can also lessen the emergence of resistance to individual or groups of antibiotics.

Finally, the results of another study indicated that the outcomes of ciprofloxacin -streptomycin combination with cefotaxime represented a synergic impact on MDR *P. aeruginosa* and a noteworthy lessening in the MIC value at a ratio of 1:3 for 20 strains with the percentage of 95.23% (163). Thus, synergistic results of cefotaxime or streptomycin– ciprofloxacin make this combination beneficial. However, more studies of *P. aeruginosa* in a clinical setting are required to assess this combination's interactions.

### Synergism of ciprofloxacin with other fluoroquinolones

As mentioned, the efflux pump is one of the main resistance mechanisms of bacteria against CIP. In this concept, Pankey et al. proposed that gatifloxacin, an 8-methoxyfluoroquinolone, could boost CIP's efficacy by inhibiting the efflux pump. Synergy testing was performed by E-test and time-kill assay for 31 clinically one kind, plasmid DNA distinct, *P. aeruginosa* segregates. Based on the E-test method, ciprofloxacin and gatifloxacin combination demonstrated synergy in six (19%) out of 31 *P. aeruginosa* isolates utilizing a summation fractional inhibitory concentration (FIC) of ≤0.5 for synergy. Also, the time-kill assay illustrated synergy for 13 (42%)/31 isolates^*^. Hence, it seems gatifloxacin inhibits the efflux pump and increases the efficacy of ciprofloxacin against *P. aeruginosa*; however, *in vitro* synergy by ciprofloxacin plus gatifloxacin against MDR *P. aeruginosa* should be evaluated in clinical setting.

### Synergism of ciprofloxacin with cephalosporins

The combination use of cephalosporins with different FQ such as ciprofloxacin was considered by researchers. Mayer et al., reported that the combination of ciprofloxacin, ofloxacin, and pefloxacin with ceftazidime, examined by disc diffusion method, demonstrated synergy for only 3–5 isolates^*^. The three ciprofloxacin-β-lactam combinations, including ciprofloxacin + ceftazidime, ciprofloxacin + aztreonam, and ciprofloxacin + azlocillin, were evaluated against MDR isolates of *P. aeruginosa*. The frequency of synergy was subordinate to antibiotic susceptibilities. Based on the evidence, in case the organism was resistant to ciprofloxacin, synergy was found in more than 50% of the isolates, but if the organism was resistant to the β-lactam (excluding ceftazidime), synergy was commonly observed in <10% of the isolates^*^.

### Synergism of ciprofloxacin with carbapenems

The combination of meropenem and ciprofloxacin seems more effective than either antibiotic alone in ICU infections due to *P. aeruginosa* strains. An earlier study examined 32 nosocomial-acquired *P. aeruginosa* strains between April 2001 and November 2001. Following the combination of ciprofloxacin with meropenem, an FIC index proposed synergy in two (6.2%) strains. The first strain was susceptible to ciprofloxacin but resistant to meropenem and imipenem; however, the second strain was both ciprofloxacin and carbapenems susceptible. Synergistic activity utilizing ciprofloxacin and imipenem happened in only one (3.1%) strain, which was susceptible to ciprofloxacin and imipenem^*^.

Time-kill synergy trials suggested that at 24 h, the sub-inhibitory meropenem and ciprofloxacin concentrations of 0.06–128 and 0.03–32 mg/L, respectively, indicated synergy against 34/51 *P. aeruginosa* strains, but that of 0.25–2 and 0.12–16 mg/L, respectively showed synergy against 18/52 *Acinetobacter baumannii* strains at the same period (164).

Rees et al. assessed bacterial killing and resistance suppression by combining meropenem with ciprofloxacin against *P. aeruginosa* in isolates collected from CF patients. Monotherapy with either meropenem or ciprofloxacin had a failure to suppress bacterial regrowth and the resistance of a hyper mutable clinical CF isolate at a high inoculum. However, the combination of 6 g of meropenem with 1.2 g of ciprofloxacin daily, both given periodically, achieved synergistic killing and resistance suppression over 8 days (165).

In another investigation, the authors separated two strongest extensive drug-resistant strains of *P. aeruginosa*, VIT PC 7 and VIT PC 9, from diabetic foot ulcer patients and tested their various resistance models utilizing whole genome sequencing. Susceptibility studies were applied using broth microdilution assay showing the impact of meropenem/ciprofloxacin susceptibility at higher concentrations, paving the way to design combinational drug examinations against these extensive drug resistance strains. The drug influence was significantly superior when the meropenem was utilized in combination with ciprofloxacin against VIT PC 7 and VIT PC 9, representing the increase of drug susceptibility by fourfold and eightfold (166).

In study performed by Pankuch et al. ciprofloxacin-meropenem combination was tested against 40 strains of *A. baumannii*. The micrograms per milliliter (MICs) of the antibiotics alone were as follows: ciprofloxacin 0.06–256 and meropenem 0.12–256. Ciprofloxacin plus meropenem, at 3 h, yielded synergy at sub-inhibitory concentrations (MICs) of ciprofloxacin (0.12 to 0.25) and meropenem (0.25) for two strains. At 24 h, the antibiotics indicated synergy against 18 strains at sub-inhibitory ciprofloxacin and meropenem concentrations of 0.12–16 and 0.25–2 μg/ml, respectively (164).

In other study by Lu et al. the *in vitro* antibacterial activity of meropenem combined with ciprofloxacin, was tested against clinically isolated XDR *A. baumannii*. The main actions of ciprofloxacin combined with meropenem were additive (56%) and indifference (44%) with synergistic and antagonistic effects (167). In the study of Sun et al. time-kill assay and checkerboard assay were conducted to study the combination effects *in vitro*. There was only one strain of *A. baumannii* for which ciprofloxacin plus meropenem indicated synergistic effect (168).

About *Enterobacteriaceae* spp. for example, in study performed by Ramadan et al. meropenem– ciprofloxacin combination showed indifferent effect (*n* = 52, 100%) on all carbapenem-resistant *K. pneumoniae* isolates, while meropenem–colistin combination indicated 25% synergism, and 59.6% indifference (169). Also in the study of Karki et al. the extensively drug resistant (XDR) isolates were tested for antimicrobial synergy and the results were interpreted as additive, synergistic, indifferent or antagonistic determining fractional inhibitory concentration (FIC) of the antibiotics. These isolates comprised *E. coli, K. pneumoniae, Acinetobacter baumannii*, and *P. aeruginosa*. All of the XDR isolates indicated “indifference” to the combination of meropenem- ciprofloxacin whereas few isolates indicated “antagonism” when tested with amikacin- ciprofloxacin and meropenem-colistin (170).

### Synergism of ciprofloxacin with other antibiotics

A combination of colistin with either of tobramycin or ciprofloxacin has displayed synergism (45.45%; five out of 11 isolates) against MDR *K. pneumoniae* isolates (171). The isolates' MICs ranged from 0.25 to 32 μg ml^−1^ for fosfomycin and from 1 to 1,024 μg ml^−1^ for CIP. The combination of fosfomycin with ciprofloxacin reflected 6% synergy on biofilm formation by MDR urinary isolates of *E. coli*. The combination also diminished the MIC of each antibiotic (22).

Synergistic interplays (interplays indices 0.69–0.83; *P* < 0.05) were found between amphotericin B (0.07–0.31 mg/L) and either ciprofloxacin (0.19–7.65 mg/L) or levofloxacin (0.41–32.88 mg/L) against *Candida albicans* and *Aspergillus fumigatus*. Synergy (interplays indices 0.56–0.87; *P* < 0.05) was also discovered between voriconazole (0.09–0.14 mg/L) and ciprofloxacin (0.22–11.41 mg/L), as well as between caspofungin (8.94–22.07 mg/L) and levofloxacin (0.14–5.17 mg/L) against *A*. *fumigatus*. Ciprofloxacin could elevate the activity of antifungal agents against both *C*. *albicans* and *A*. *fumigatus* (172).

*In vitro* and *in vivo* examinations have highlighted that tigecycline in combination with ciprofloxacin is a powerful choice for treating invasive *Vibrio vulnificus* infection. An *in vitro* time-kill assay manifested synergism between tigecycline and ciprofloxacin. The survival rate was remarkably higher in mice treated with tigecycline plus ciprofloxacin than those treated with cefotaxime plus minocycline. Vancomycin-ciprofloxacin combination can be synergic against enterococci resistant to both vancomycin and ciprofloxacin. Still, it would be unlikely to have any excellence in treating enterococcal infections due to the high concentrations needed (173).

### Synergism of ciprofloxacin with nanoparticles

Nanoparticles (NPs), particles with a size of 1–1,000 nm (commonly 5–350 nm in diameter), are made of any biocompatible substance. Different studies have reported acceptable antibacterial activity for NPs even against biofilm community of bacteria (174). To this end, the combined use of NPs with different antibiotics such as ciprofloxacin was considered by researchers for inhibition of bacterial growth and elimination of the biofilm community of these microorganisms.

In this concept, in the recently published study the authors synthesized embelin (Emb, isolated from *Embelia tsjeriam-cottam*)-chitosan-gold NPs (Emb-Chi-Au) were evaluated for their potential synergistic activity with ciprofloxacin by checker boarding assay and time-kill curve analysis. The NPs diminished the MIC of ciprofloxacin by 16- and 4-fold against MDR *P. aeruginosa* and *E. coli* strains, respectively. Furthermore, FIC records with ≤0.5 values affirmed the synergy between the ciprofloxacin and Emb-Chi-Au NPs, further confirmed at ½ MICs in both *P. aeruginosa* and *E. coli*, using time-kill curve analysis. In addition, Emb indicated the efflux pump-inhibitory potentials against both the organisms under consideration. Hence, the synergistic application of ciprofloxacin with Emb-Chi-Au NPs showed inhibitory impacts on two of the most MDR bacteria. To this end, the authors proposed that the inhibition of bacterial efflux pumps by NPs must have retained the concentrations of ciprofloxacin inside the cell, which acted against bacterial DNA topoisomerase/gyrase (175).

In addition to mentioned NPs, AgNPs are used in different studies to enhance ciprofloxacin efficacy. In one of these studies, the authors surveyed the synergistic bactericidal impact of AgNPs and ciprofloxacin on different bacteria such as *Pseudomonas solanocearum, Pseudomonas syringae, Xanthomonas malvacearum*, and *Xanthomonas campestris*. When 0.2 mM of AgNPs were combined with 1 μg of ciprofloxacin, the antiphytopathogenic activity was surprisingly expanded to 36, 40, 33, and 35 mm against all the mentioned bacteria, respectively. Similarly, MIC and minimum bactericidal concentration (MBC) values were diminished significantly, indicating the synergistic activity between AgNPs and ciprofloxacin (176).

In line with these results, Nikparast et al. reported that the combined antibacterial activity of ciprofloxacin with AgNPs declined the MIC of antibiotics from 0.125 to 0.0625 μg/ml toward *P. aeruginosa*. Ciprofloxacin MIC against *P. syringae* reduced from 0.25 to 0.0625 μg/ml in combination with 6.25, 12.5, and 25 μg/ml of AgNPs (177).

In addition to AgNPs, zinc oxide (ZnO) was another metal-NPs that was used in combination with ciprofloxacin for enhancement of antibacterial activity. To this end, the authors synthesized ZnONPs, functionalizing them by Glu and conjugating them with thiosemicarbazid (TSC) to increase their efficacy against ciprofloxacin -resistant *S. aureus*. The results showed the synergistic activity of ciprofloxacin and synthesized NPs against ciprofloxacin -resistant *S. aureus*. Thus, the authors introduced ZnO@Glu–TSC NPs as a promising new antibacterial agent for therapeutic and preventive purposes (178).

The exact interaction of metal-NPs and ciprofloxacin has not been reported yet. However, it seems that these NPs, after attachment to the bacterial cell membrane, lead to the formation of gap on the bacterial cell walls, and damage to the cell membrane, thereby allowing the ciprofloxacin to enter the periplasm of the bacterial cells. Therefore, the combination of ciprofloxacin and metal NPs yield novel antimicrobial agents with synergistic properties that could be exploited for higher antibacterial activity. However, due to the high toxicity of these NPs for human cells, further investigation needs to be performed to evaluate the safety of these NPs for medical applications.

**Figure 4:**
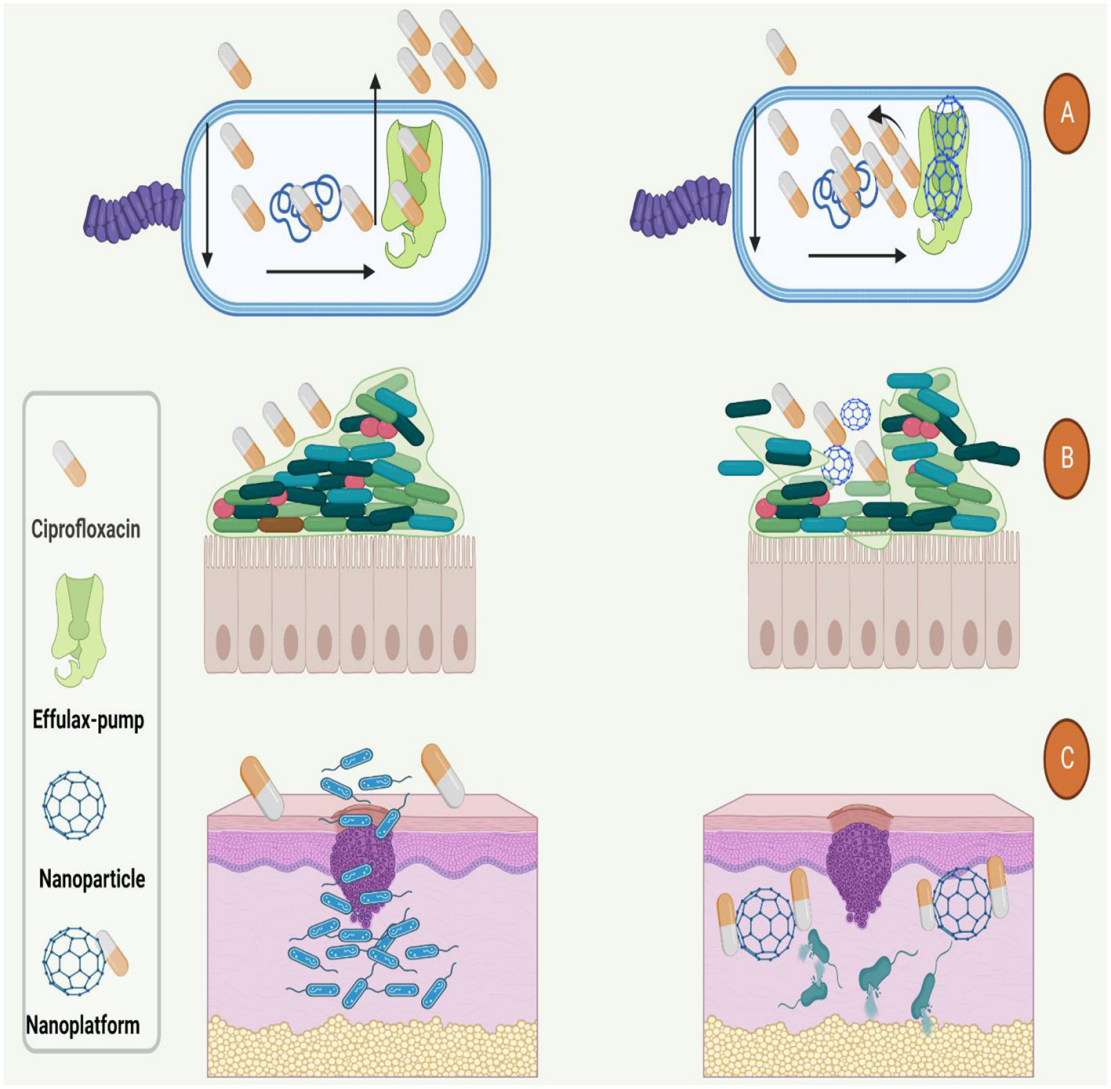
Used nano-platforms for enhancement of ciprofloxacin efficacy against bacteria. **(A)** Nanoparticles could boost the antibacterial function of ciprofloxacin by inhibition of efflux-pumps. **(B)** Nanoparticles increase the antibacterial activity and penetration of ciprofloxacin to the **(B)** dipper layers of biofilm and **(C)** body organs such as the skin.

It's noteworthy to mention that, other studies that have used of nanoplatforms for enhancement of ciprofloxacin efficacy are presented in Table 4. Based on this table and mentioned studies, ciprofloxacin delivery can be modified by encapsulating with or incorporating different polymeric NPs such as poly lactic-co-glycolic acid (PLGA), chitosan, arginine, albumin, and other organic and inorganic nanostructure systems (179). Furthermore, studies have also shown that nano-platforms could enhance the efficiency of ciprofloxacin against bacterial cells, interfere with the biofilm community, enhance the penetration and protect the drug from deactivation or efflux (Figure 4).

**Table 4:** The studies have used nano-platform for the enhancement of ciprofloxacin against different bacteria.

**References**	**Nanoplatforms for delivery of ciprofloxacin**	**Bacteria**	**Outcomes**
(180)	Ciprofloxacin-AgNPs	*A. baumannii* *S. marcescens* *S. aureus*	Compared to ciprofloxacin alone, this compound showed better antioxidant, anti-biofilm, and antibacterial function against the pathogenic bacteria tested
(181)	Chitosan/dysprosium oxide	NA	This nanocomposite has good potential for a controlled drug delivery system
(182)	Synthesized red blood cell membrane-coated PLGA	*K. pneumoniae*	This NP showed good antibacterial and anti-infection ability
(183)	Gelatin-sodium carboxymethyl cellulose composite nanogels	*S. aureus*	This compound showed antibacterial activity with sustained-release performances
(184)	Nano-fluid containing carbon nano-tubes	*Drug-resistant K. pneumoniae*	Simultaneous usage of nano-fluid and antibiotics could enhance antibiotic effectiveness at lower doses
(185)	Hemicelluloses from *Lallemantia royleana*, chitosan/chitin and glutaraldehyde	*S. aureus* *E. coli*	This compound showed comparable activity against *E. coli* to that of ciprofloxacin and relatively lower activity in the case of *S. aureus*
(186)	Graphene-silk fibroin macromolecular hydrogel dressings	*S. aureus* *P. aeruginosa*	This compound improved antibacterial activity against both bacteria and burn wound infection
(187)	Clay/alginate/imidazolium-based ionic liquid	*E. coli* *P. aeruginosa*	Ciprofloxacin-loaded nanocomposites showed significantly higher antibacterial activity in comparison with free ciprofloxacin
(188)	Hyaluronic acid functionalized self-nano-emulsifying drug delivery system	*Salmonella typhi*	The drug-delivery system with ciprofloxacin showed an improved ability to permeate goat intestinal mucus, antibiofilm activity, and oral pharmacokinetics compared to free ciprofloxacin
(189)	Ciprofloxacin-azithromycin NPs on chitosan nanocarriers	*P. aeruginosa*	This compound significantly inhibited the biofilm community of bacteria in comparison to the free ciprofloxacin
(190)	Chitosan microspheres/nano hydroxyapatite- titanium	*S. aureus*	Showed antibacterial activity
(191)	Citric acid cross-linked carboxymethyl guar gum nanocomposite films	NA	Enhanced the wound healing
(192)	Sodium alginate cross-linked with nano-hydroxyapatite	*P. aeruginosa* *S. aureus* *E. coli*	Showed antibacterial, especially against *S. aureus*
(193)	Poly(DL-lactide-co-glycolide) NPs	*P. aeruginosa* *S. aureus*	The NPs were safer and more effective against bacteria in comparison to free drugs
(194)	poly(vinyl alcohol) /citric acid/Ag NPs	*S. aureus* *E. coli*	Showed an effective antibacterial activity.
(195)	Fe_3_O_4_@ polyacrylic acid @ZIF-8	*S. aureus* *E. coli*	This compound decreased the growth of bacteria
(196)	Zn containing mesoporous silica nanospheres into polycaprolactone electrospun fibers	*E. coli*	Showed antibacterial and wound healing capacity
(197)	Cerium-doped nano-bioactive glasses	*P. aeruginosa* *S. aureus* *E. coli* *Bacillus subtilis*	Showed antibacterial activity against all studied bacteria
(198)	Nano gold embedded cellulose grafted polyacrylamide nanocomposite hydrogel	*E. coli* *Shigella flexneri* *Bacillus cereus* *Listeria Inuaba*	This nanocomposite with improved rheological and thermal characteristics is suitable and proposed as a good carrier for *in vitro* release of ciprofloxacin drugs

NPs, nanoparticles; NA, not applicable; NR, not reported.

### Synergism of ciprofloxacin with natural products

Recent studies indicate that new antimicrobial agents are required to reduce the toxicity of conventional antimicrobial agents. Furthermore, combination therapy could improve the efficacy of different antimicrobials (199). In this regard, the combined ciprofloxacin and different natural products were considered to inhibit bacterial growth.

The recently published study used the checkerboard microdilution and evaluated *in vitro* interaction between *Thymbra spicata* L. extracts and certain antibiotics such as amikacin, cefotaxime, ampicillin, and ciprofloxacin against MDR *K. pneumonia* and *S. aureus*. The combination of amikacin, cefotaxime, and ampicillin plus plant extraction showed synergistic activity against S. *aureus*. In contrast, the joint activity of plant extract with ciprofloxacin indicated indifferent and additive activity. Furthermore, ciprofloxacin showed an indifference and additive effect with sensitive and resistant *K. pneumoniae* strains when combined with all *T. spicata* extracts (200).

Based on the checkerboard synergy technique, nbutanolic *Cyclamen coum* extract in combination with ciprofloxacin represented a synergistic effect against *P. aeruginosa* biofilms (ΣFBIC = 0.496) (201). The extricates of four customarily utilized therapeutic plants, i.e. *Plumbago zeylanica* (root), *Hemidesmus indicus* (stem), *Acorus calamus* (rhizome), and *Holarrhena antidysenterica* (bark), were examined against the clinical isolates of MRSA and methicillin-sensitive *S. aureus, P. zeylanica* and *H. antidysenterica* demonstrated synergism with ciprofloxacin (202).

Additionally, the MIC findings of another investigation uncovered that combinatorial impacts of Sami-Hyanglyun-Hwan ethanol extract (SHEE) with ciprofloxacin had 2–32-fold reduction in concentration as those needed by SHHE alone. The antibacterial activity of SHHE obviously declined the MICs of ciprofloxacin against *S. aureus* strains. The checkerboard method suggested that the combinations of SHHE with ciprofloxacin had a partial methicillin-resistant synergistic or synergistic impact on MRSA. The time-kill curves also proved that *S. aureus* in combination with SHHE and ciprofloxacin treatment, lessened the bacterial counts significantly after 24 h (203). Chrysoeriol had a notable synergistic impact when combined with ciprofloxacin and oxacillin against epidemic methicillin-resistant *S. aureus* 15 (EMRSA-15) and EMRSA-16, respectively, both of which are the UK epidemic MRSA strains (204). When biochanin A (BCA) was combined with ciprofloxacin, the FIC index data exhibited that there was synergy in all 12 of the *S. aureus* strains examined. The outcomes of time-kill tests and agar diffusion tests affirmed synergy between BCA and ciprofloxacin against *S. aureus* strains. These results proposed that BCA can be combined with FQs to produce a potent antimicrobial agent (205).

On the other hand, the results of another study showed that the combination of Propolis, a mixture of a complex chemical composition containing essential oils, balms, pollen, minerals, vitamins, and proteins, with ciprofloxacin has shown an antagonistic effect against MRSA. The ciprofloxacin action is diminished when combined with Propolis. In five of the seven strains studied, further growth of MRSA was found in combinations in concentrations of each substance separately applied. Hence, the combination of both substances is noxious (206).

Thus, combining ciprofloxacin with natural products could lead to several advantages such as boosted potency, a reduced dose of drugs needed and minimized toxicity, which ultimately helps inhibit different bacteria even MDR isolated. Although, the exact mechanism by which natural products synergizes with ciprofloxacin was not investigated in the studies mentioned above. Therefore, additional molecular and *in vivo* studies are needed to confirm the practical utility of these combinations. Finally, in addition to natural products, the combined use of ciprofloxacin with various natural compounds, which have antimicrobial properties, such as curcumin, eugenol, cinnamomum, and carvacrol, should be considered.

### Synergism of ciprofloxacin with photodynamic/laser therapy

Photodynamic therapy (PDT) has been identified as an effective treatment for the inhibition of bacterial infections such as *E. faecalis* infection in root canal dentine (207). In this method, a specific wavelength excites a photosensitizer, photoactive dye, and leads to the generation of singlet oxygen or other reactive oxygen species (ROS) that can eliminate the target bacteria (208). Methylene blue (MB), due to various characteristics such as low molecular weight and toxicity in mammalian cells, and hydrophilicity, are reported as a potential photosensitizer for PDT (209). In recent years, different methods have been used to enhancement of PDT efficacy for the inhibition of bacterial infections. The use of antibiotics in combination with PDT is one of these methods. In this regard, researchers used ciprofloxacin to boost the performance of the PDT.

To this end, the findings of the recently published study showed that *S. aureus*, even with the lowest ciprofloxacin and MB concentrations (0.0625 and 6.25 μg/mL, respectively), bacterial killing was remarkably developed when compared to MB–PDT alone for the exact light dose. The best findings were achieved after the combination treatment of PDT with, followed by ciprofloxacin on biofilms, which enhanced bacterial diminishment on biofilms, resulting in a 5.4 log diminishment for *S. aureus* biofilm and approximately seven logs for *E. coli* biofilm (210). In another investigation also, the authors reported that essential oil obtained from *Eugenia jambolana* interferes with the action of antibiotics against bacteria exposed to LED lights. This trial showed that irradiation of *E. coli* and *S. aureus* with blue or red light in the presence of ciprofloxacin is more beneficial than antibiotic monotherapy (211). Therefore, PDT can destroy the bacterial community using various possible mechanisms such as interference with cellular hemostasis and membrane permeability, modulation of DNA and RNA synthesis, and alkalization of the cytoplasm and cell membrane depolarization. In this regard, the combine use of PDT and ciprofloxacin can be considered for treatment of bacterial infections especially infection that caused by MDR bacteria; however, the data about this kind of treatment is very limited and more confirmatory studies are needed.

### Synergism of ciprofloxacin with bacteriophages

**Figure 5:**
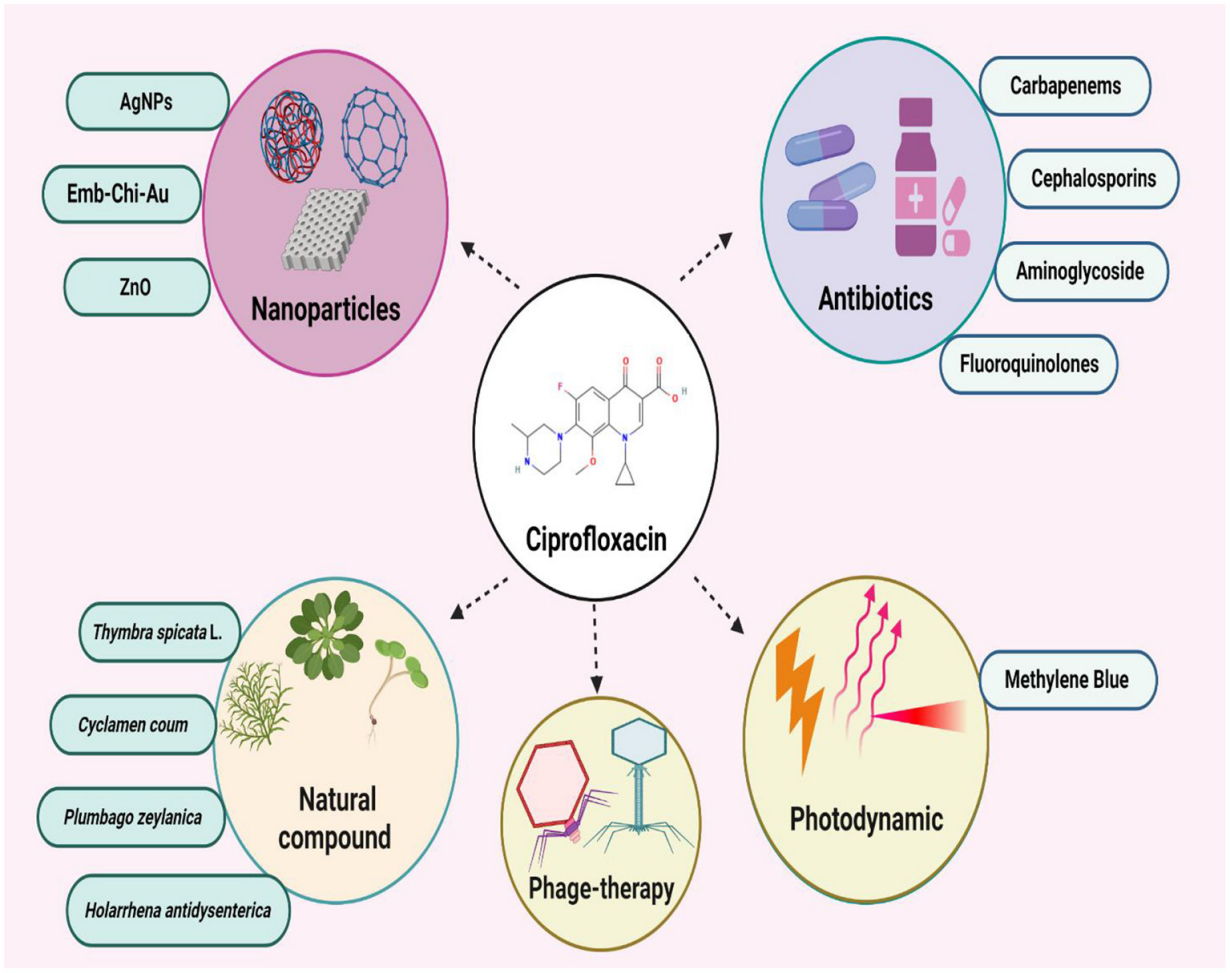
The combination uses ciprofloxacin and other antibacterial agents.

Bacteriophages (phages), viruses that infected bacteria, were first discovered in the middle of the 20th century, and due to their great function in the inhibition of MDR bacteria, was considered by scientist as non-antibiotic approaches for the treatment of bacterial infections. Eukaryotic cells have no receptors for phages; therefore, they can be used to treat bacterial infections (212). A phage cocktail containing two or more bacteriophage mixtures with different host ranges in a single suspension could lead to a better antibacterial effect than single phage therapy (213, 214). Phages could penetrate the dipper layer of biofilm and damage its structure by producing natural enzymes. Additionally, endolysins are produced at the end of the lytic cycle of the phages. This enzyme could destroy bacterial cell walls by the cleavage of peptidoglycan (215, 216). To this end, combination therapy of antibiotics and phage not only causes a reduction in the number of bacteria but also can be related to the management of phage-resistant bacteria levels (217). Therefore, this section will discuss the combination therapy of ciprofloxacin and phage for treating bacterial infections (Figure 5).

Recently published studies reported additive or synergistic effects of ciprofloxacin and phage combination (134, 218–223). Gurney et al. reported that phages could interact with different structures of *P. aeruginosa*, such as lipopolysaccharide structure (LPS) and the efflux pumps. Therefore, phages could increase the permeability of bacteria and the drug dosage by inhibiting efflux pumps (134). Another study also indicated that combining phage cocktail and ciprofloxacin could increase the number of MDR *P. aeruginosa* strains' susceptibility to this antibiotic. This combination therapy also resulted in the re-sensitization of *P. aeruginosa* to ciprofloxacin. Noteworthy, the animal wound model result showed that phage-only treated mouse wounds had mutations for phage receptors; thus, these animals were resistant to infection with phage. However, these mutations were not detected in the combination treatment bacteria, suggesting that the treatment with phages and antibiotics reduced the incidence of the bacteria becoming resistant to the phage treatment (218). In another investigation, the authors reported that intratracheally treating mice (with acute lung infection) with phage- ciprofloxacin combination powder remarkably decreased the bacterial load in the lungs. In contrast, single treatments failed to reduce the bacterial count (221).

Therefore, the combination use of phage–antibiotic is a promising approach for in inhibition of MDR bacteria, especially *P. aeruginosa*. The phage- ciprofloxacin synergistic effect in killing bacterial cells could be due to a selective pressure under which the bacteria mutate in one trait to improve fitness while suffering a decrease in another trait. A recently published study reported an evolutionary trade-off effect when phage treatment imposed a selective pressure on MDR bacteria. When bacteria lose their receptor for phage binding, they resist to infection by phages. However, in this condition, bacteria regained sensitivity to a different antibiotic, such as ciprofloxacin. Another possible reason might be morphologic changes of bacterial cells when exposed to sub-inhibitory concentrations of antibiotics. In this circumstance, antibiotic exposure led to the elongation of bacterial cells but did not divide, which could improve phage assembly and maturation (221, 224, 225).

Additionally, as mentioned in previous parts of the manuscript, the biofilm community of bacteria is one of the most important challenges in treating infection. Given that, the combination uses of ciprofloxacin and phages have been considered by scientists for the elimination of bacterial biofilm. Tkhilaishvili et al. reported that a higher concentration of ciprofloxacin is required to suppress the growth of dual-species biofilms compared to monospecies biofilms. On the other hand, combining phages with ciprofloxacin significantly enhanced the anti-biofilm activity of both antimicrobials with complete eradication of *S. aureus*/*P. aeruginosa* biofilms (226). In line with these findings, a recently published study also reported that antibiotics such as ciprofloxacin and phages alone had a modest effect in killing bacteria in biofilm community.

Nonetheless, when these compounds were used at the same time, especially when ciprofloxacin was added sequentially after 6 h of phage treatment, a significant enhancement in the killing activity was detected (227). It seems phages *via* depolymerases could degrade the biofilm matrix, consequently enhancing antibiotic penetration into the deeper layers of the biofilm (227–229). However, depolymerases was not detected in some phages; therefore, the mentioned phenomenon might not have been responsible for the synergistic action of the phages and antibiotics combined therapy. In these cases, it's possible that phages using of biofilm void spaces could access the dipper layers of the biofilm. Afterward, phages replicate in the biofilm's deeper layer and interrupt the biofilm's extracellular matrix. The addition of antibiotics following this interruption causes an improved bacterial killing due to the deeper penetration of phages and antibiotics (227, 230).

Taken together, combining ciprofloxacin with phages can be synergistic in destroying the bacteria in the biofilm community; hence, this combination therapy is a promising candidate for treating infections are caused by MDR bacteria. However, some important challenges, such as the time of antibiotic application, the concentration of antibiotics, and the exact interaction of phages with eukaryotic cells, should be evaluated in further studies.

Finally, its noteworthy that recently published studies that have used various antibacterial agents to enhance ciprofloxacin efficacy against different bacterial infections in animal models and *in vivo* studies are presented in Table 5.

**Table 5:** Studies have used various approaches to enhance ciprofloxacin activity against bacterial infection in animal models and *in vivo*.

**References**	**Antibacterial agents**	**Animal models**	**Bacteria**	**Outcome**
(231)	Recombinant glycoside hydrolases	Lung infection	*P. aeruginosa*	The Co-T^*^ leads to a greater reduction in pulmonary bacterial burden than with either agent alone
(232)	PDT with cationic imidazolyl photosensitizers	Wound infection	*E. coli*	This synergic combination decreased the ciprofloxacin and photosensitizer needed for full bacteria inactivation
(233)	Toll-like receptor 2 agonist	*B. anthracis* infected mice	*B. anthracis*	The Co-T showed augmented activity in protecting mice from infection
(234)	Non-hydroxamate LpxC inhibitor	Murine model of pneumonia	*K. pneumoniae*	The Co-T decreased the production of IL-6 and LPS release induced by ciprofloxacin in the lung
(235)	Macrophage-membrane NPs	Mouse peritoneal infection model	*S. aureus*	NPs killed staphylococci more effectively than ANPs without membrane encapsulation
(236)	Neutrophil-factor S100A8/A9	Biofilm-infected chronic wounds	*P. aeruginosa*	Ciprofloxacin monotherapy developed resistance (after 14 days), while combination therapy changed the resistance pattern
(237)	Ciprofloxacin/rolipram nanostructured lipid carriers	Bacteremia with organ injury	MRSA	This compound remarkably reduced elastase distribution and MRSA burden in the organs of MRSA-infected animals
(238)	Thymine	Galleria mellonella infection model	*E. coli*	Thymine significantly enhanced ciprofloxacin activity
(221)	Phage	Neutropenic mouse model of acute lung infection	*P. aeruginosa*	The Co-T remarkably decreased the bacterial load in mouse lungs. In contrast, no significant reduction in the load of bacteria was detected when the animals were treated only with phage or ciprofloxacin
(239)	Truncated alpha-defensins analog 2Abz23S29	Murine model of urinary tract infection	UPEC	The macrophage inflammatory protein/2 and IL-6 in infected mice treated with combination therapy were remarkably higher than in the untreated mice
(240)	Antibiotic-loaded adipose-derived stem cells	Rat implant-associated infection model	*S. aureus*	Rats treated with combination therapy had the lowest abscess formation, modified osteomyelitis scores, and bacterial burden on the implant
(241)	PLGA microsphere-based composite hydrogel- ginsenoside Rh2	Mouse model of MRSA skin infections	MRSA	Great potential for the treatment of wound infection
(242)	2-(2-aminophenyl) indole (efflux pump inhibitor)	Murine thigh infection model	*S. aureus*	The Co-T indicated significant efficacy against bacterial infection
(173)	Antibiotics	Invasive infection	*Vibrio vulnificus*	The survival rate was significantly higher in mice treated with tigecycline plus ciprofloxacin than in mice treated with cefotaxime plus minocycline
(243)	Glycyrrhizin	Ocular infection	*P. aeruginosa*	The Co-T vs. ciprofloxacin remarkably decreased plate count, clinical scores, and myeloperoxidase
(244)	3-hydroxypyridin-4-one chelator	Pneumonia	*Acinetobacter baumannii*	Treatment with ciprofloxacin alone was insufficient for removing infection caused by ciprofloxacin-resistant bacteria; however, the combination therapy significantly improved treatment efficacy
(245)	Immunomodulatory S100A8/A9	Murine chronic wound model	*P. aeruginosa*	Augmented the effect of ciprofloxacin

PDT, photodynamic therapy; Co-T, combination therapy; LPS, lipopolysaccharide; NPs, nanoparticles; MRSA, methicillin-resistant Staphylococcus aureus; UPEC, uropathogenic E. coli.

* Combination therapy of antibacterial agent with ciprofloxacin.

## Conclusion

Ciprofloxacin's potential for the treatment of a large spectrum of bacterial infections led to the overuse of this drug in clinical practice and developed alarming levels of ciprofloxacin resistance as a consequence of heavy use. To preserve this beneficial agent, prescribers must ensure that ciprofloxacin is a proper choice and administer enough doses to limit the risk of selecting resistant mutant bacterial subpopulations. The increasing incidence of ciprofloxacin-resistant pathogens jeopardizes the continued empiric use of ciprofloxacin and raises the urgent need to develop novel ciprofloxacin derivatives potent against both drug-susceptible and drug-resistant pathogens and discover useful synergism between ciprofloxacin and other antibacterial agents. As mentioned earlier in this study, recent studies have reported a wide range of synergism between ciprofloxacin and other antibacterial agents. Therefore, the combination use of ciprofloxacin and other antibiotics and antibacterial agent should be considered in future studies because combination therapy could increase antibacterial performance of ciprofloxacin especially against MDR strains.

## Author contributions

SK and MH conceived and designed the study. AS, MAr, MK, MAb, MG, MH, and SK contributed in comprehensive research. AS and MH participated in editing the manuscript. All authors have read and approved the manuscript.
